# The Association between Food Groups, Nutraceuticals, and Food Supplements Consumption on Vascular Health Outcomes: A Literature Review

**DOI:** 10.3390/life14091210

**Published:** 2024-09-23

**Authors:** Xenophon Theodoridis, Michail Chourdakis, Androniki Papaemmanouil, Stavroula Chaloulakou, Niki Papageorgiou, Athina Vasiliki Georgakou, Georgios Chatzis, Areti Triantafyllou

**Affiliations:** 1 Laboratory of Hygiene, Social and Preventive Medicine and Medical Statistics, School of Medicine, Faculty of Health Sciences, Aristotle University of Thessaloniki, University Campus, 54124 Thessaloniki, Greece; xtheodoridis@auth.gr (X.T.); acpapaem@auth.gr (A.P.); chstav@auth.gr (S.C.); npapaga@auth.gr (N.P.); georathi@auth.gr (A.V.G.); 2 Third Department of Internal Medicine, Papageorgiou Hospital, Aristotle University of Thessaloniki, 56429 Thessaloniki, Greece; 3 School of Physical Education and Sports Science, Aristotle University of Thessaloniki, 57001 Thessaloniki, Greece; dietchatzis@gmail.com

**Keywords:** vascular health, arterial stiffness, endothelial function, food groups, nutraceuticals, supplements

## Abstract

Vascular aging, marked by alterations in the structure and function of blood vessels, including heightened arterial stiffness and impaired endothelial function, is linked to a higher likelihood of developing cardiovascular and age-associated pathological conditions. Oxidative stress and inflammation are key stimulation factors in vascular aging. Engaging in healthy dietary habits could enhance the functioning of blood vessels. The aim of this study was to conduct a literature review of the evidence regarding the relationship between food regimens, nutraceuticals, and dietary supplements and vascular health. A search of electronic databases, including PubMed, Scopus, and Web of Science Core Collection, was performed. Experimental and observational studies evaluating the association between food groups, nutraceuticals, supplements, and endothelial function and/or arterial stiffness were deemed eligible for this narrative review. Based on the current body of the included studies, food groups, nutraceuticals, and dietary supplements may not demonstrate superiority over placebos in enhancing markers of vascular health. To obtain more reliable evidence on the effectiveness of interventions in vascular health, additional RCTs with larger sample sizes, extended follow-up periods, and multi-center participation are necessary. Enhancing the credibility of these RCTs requires better control of dietary variables and more precise measurement of vascular health markers.

## 1. Introduction

Aging refers to the biological process characterized by the aggregation of numerous types of molecular and cellular impairment over time. This results in a steadily declining level of a person’s abilities, as well as an increasing risk of disease and eventually death. Aging is a growing concern, with the global population over 60 expected to reach 2.1 billion by 2050 [1]. Attenuation of vascular function, specifically endothelial dysfunction and stiffness of large elastic arteries, forms an important association between aging and cardiovascular risk. Research has shown a gradual decrease in arterial function over the course of time [2].

The endothelium is essential for preserving vascular homeostasis, preserving the balance between vasodilation and vasoconstriction [3]. Vascular aging alters the arteries’ structure and function [4]. Vascular age could be roughly estimated by systolic blood pressure and pulse pressure [5]. One of the principal assessment methods of endothelial dysfunction is flow-mediated dilatation (FMD) [6]. On the other hand, the difference between the biological and chronological ages of major arteries is mirrored by arterial stiffness (AS) [7]. Pulse wave velocity (PWV) and the augmentation index (AIx) are the most common methods for the evaluation of arterial stiffness [8,9].

Oxidative stress and inflammation are key stimulation factors in vascular aging. An excess of reactive oxygen species (ROS), including superoxide and hydrogen peroxide, are produced by aging vasculature and contribute to the reduction of the vasodilatory action of nitric oxide (NO), as well as in the increase of the generation of peroxynitrite. Furthermore, diminished antioxidant response mediated by erythroid-2-related factor-2 (Nrf2) and the downregulation of mitochondrial manganese superoxide dismutase (SOD2) take part in the development of chronic oxidative stress in aging arteries. This is associated with a chronic low-grade inflammatory phenotype that contributes to impaired endothelial vasodilation. Nuclear factor-κB (NF-κB) upregulates in the vascular cells of elderly individuals, leading to a proinflammatory response to oxidative stress. Chronic NF-κB activation is caused by increased angiotensin-II signaling and downregulated sirtuins, which hinders cellular response to acute ROS production [10]. Antioxidants, including those that may be found in the human diet, seem to play an important role in controlling oxidative stress [11]. Lifestyle factors, such as enhanced dietary fiber intake, diminished saturated fat intake, and moderate alcohol consumption, have also been linked to reduced inflammation [12].

Early vascular aging is also a matter of concern. As an example, according to a newly released systematic review, smoking, hypertension, and diabetes mellitus emerge as major risk factors for the observed acceleration in arterial stiffness in subjects with acute ischemic stroke [13]. Notably, the supportive role of healthy nutrition in the treatment of hypertension and diabetes mellitus is well documented [14,15]. 

Healthy lifestyle behaviors have been observed to prevent or treat vascular dysfunction related to aging [16]. Nutraceuticals, such as omega-3 fish oils, green tea polyphenols, soy isoflavones, sulforaphane, lactotripeptides, allicin, spermidine, capsaicin, shogaol, carnosol, cocoa flavonols, lycopene, quercetin, and nitrite/nitrate, are among the studied dietary components, which seem to be related to greater endothelial function (EF) and/or decreased arterial stiffness [17]. Furthermore, antioxidant vitamins, such as vitamins C and E and β-carotene, are revealed to have valuable effects on vascular endothelial function [18]. Moreover, a greater consumption of fruits and vegetables has been linked to improved endothelial function, among others [9]. However, there are also studies observing the aforementioned relationships that failed to show a beneficial effect [19]. 

Dietary recommendations, as a part of official clinical guidelines in numerous health conditions, have been gaining ground in recent years and the field of nutrition-specific scientific evidence has attracted significant scientific attention. The relationship between dietary patterns, arterial stiffness, and endothelial function has been investigated in a previous narrative review of our team [20], in which we concluded that the Mediterranean, Dietary Approaches to Stop Hypertension, and low-calorie diets may improve vascular health. Most of the published reviews evaluate the effect of dietary components on other endpoints, such as blood pressure levels. The aim of this current review is to evaluate the evidence on how specific food groups, nutraceuticals, and dietary supplements influence vascular health, including the results of the latest studies. To our knowledge, this is the first review in which evidence regarding all these dietary components is summarized. In particular, the sections of food groups, fruits and vegetables, legumes, dairy products, nuts and oils, red meat, poultry and fish, and coffee and alcohol are discussed. Moreover, the domain of nutraceuticals contains information regarding nitrate-nitric oxide, resveratrol, flavonoids, coenzyme Q10, curcumin, omega-3 fatty acids, astaxanthin, and selenium. Finally, the supplements section incorporates magnesium, vitamin B, folic acid, zinc, vitamin C, vitamin E, vitamin D, vitamin K, potassium, and multivitamin supplementation.

## 2. Materials and Methods

The primary objective of this review was to implement an extensive evaluation and synthesis of the available evidence regarding the efficacy of the use of nutraceuticals, specific food products, and vitamin–mineral supplements on vascular health, such as AS and EF. Its goal is to provide a comprehensive review and present current knowledge of how nutrition affects vascular outcomes, such as PWV, AIx, and FMD, among others.

A search of electronic databases, including PubMed, Scopus, and Web of Science Core Collection, was performed to thoroughly cover the medical literature. There were no restrictions based on publication dates in the current narrative review. The inclusion criteria for this narrative review involved peer-reviewed articles, systematic reviews, randomized controlled trials (RCTs), clinical trials (CT), and observational studies assessing the effects of the consumption of nutraceuticals, certain food products, and dietary supplements on vascular health and EF. Studies that incorporated pregnant populations, those written in languages other than English, and those published before 2000 were excluded.

## 3. Food Groups

It has been hypothesized that some dietary patterns have cardio-protective outcomes. The macro- and micro-nutrients that constitute a daily diet have a modest but significant effect on vascular health/aging [21]. Healthy lifestyle habits based on Mediterranean diet (MedDiet) adherence and regular exercise are associated with lower arterial stiffness in a metabolically healthy population with overweight or obesity (MHOe) (aged ≥65 years) [22]. Thus, in order to further evaluate potential nutrition-related health outcomes, it seems reasonable to classify dietary components into food groups and evaluate each one separately [23].

The shared denominator among various organizations’ guidelines regarding the role of nutritional interventions in preventing cardiovascular diseases and promoting vascular health is the increase in the consumption of plant-based and whole wheat products, fruits, vegetables, and fatty fish [24]. This could be attributed to the fact that fruits and vegetables are sources of phytochemical antioxidants and contribute to cardio health promotion [25].

Red meat, of which overconsumption is a component of the Western diet, seems to be linked to arterial stiffness and endothelial function [26]. Additionally, alcohol and coffee ingestion could affect endothelial function, but the available evidence is still inconclusive, as described below.

### 3.1. Fruits and Vegetables

The link between arterial health and fruit and vegetable consumption is related to the numerous nutrients and compounds found in these food groups. These nutrients appear to be effective in the mitigation of inflammation and oxidative stress in blood vessels, while dietary fiber plays a significant role in regulating cholesterol levels and promoting optimal blood pressure [26]. Also, consuming high quantities of fruits and vegetables is associated with a decrease in cardiovascular diseases due to their capacity to protect endothelial function [27].

Vegetables like leafy greens, cruciferous vegetables, legumes, and yellow-orange-red vegetables have been shown to have favorable effects on CVD risk in a number of systematic reviews and meta-analyses [28,29]. These vegetables may be beneficial to cardiovascular health due to their high composition of nutrients and phytochemicals [29,30]. A diet rich in fruits and vegetables with high potassium content, such as leafy greens, exhibits vasoactive properties and may lower blood pressure by reducing vascular smooth muscle contraction. Furthermore, potassium enhances urinary sodium excretion and decreases insulin resistance and oxidative damage. Insulin resistance, along with compensatory hyperinsulinemia and reactive oxygen species, can disrupt the homeostasis of the vascular wall and contribute to the onset of hypertension [31].

According to a systematic review of 46 clinical trials conducted by Blanch and colleagues [9], high potassium intake may improve vascular function, but there was insufficient evidence linking high fruit and vegetable intake with improved vascular outcomes.

A 12-month randomized controlled trial involving individuals with diabetes (n_intervention_ = 45; n_control_ = 47) also supported this finding [32]. The study participants were randomized to receive either dietary counseling to increase fruit, vegetable, and dairy consumption, or to follow their usual diet. According to the findings, after a year, there was no time-by-treatment effect on central blood pressure (cBP), AΙx, or carotid femoral pulse wave velocity (cfPWV). More specifically, over time, AIx (*p* = 0.002), centrally augmented pressure (*p* = 0.02), and cfPWV (*p* = 0.048) showed an increase, with no observed time-by-treatment effect. 

Possible mechanisms regarding the beneficial action of fruit and vegetables are available through experimental studies that showed that a number of natural substances possess cardiovascular-protective effects through an array of procedures, such as regulating blood pressure, optimizing lipid levels, reducing inflammation, reducing oxidative stress, and modifying gut microbiota [25,33].

Numerous studies have suggested that a variety of vegetables, among them tomatoes, potatoes, onions, celery, broccoli, lettuce, and asparagus, which contain a number of bioactive components, such as vitamins, essential minerals, dietary fibers, botanical proteins, and phytochemicals, might have merit as candidates for CVD prevention and treatment. The possible cardioprotective properties of vegetables include the reduction of blood pressure, the regulation of lipid and blood glucose metabolism, and antioxidant, anti-inflammatory, and antiplatelet properties. Additional mechanisms include enhanced endothelial function, reduced myocardial damage, and influenced related enzyme activities, gene expressions, and signaling pathways among other biomarkers correlated with CVD risk [34,35,36].

A RCT that recruited 38 normotensive participants, randomized to follow either a high-nitrate (<300 mg/day from green leafy vegetables), or low-nitrate diet for 7 days, reported that although the high-nitrate diet intervention resulted in at least a fourfold increase in salivary and plasma nitrate and nitrite (*p* < 0.001), arterial stiffness, as measured using cfPWV and AΙx, showed no significant changes [37].

In a placebo-controlled crossover design, 27 healthy participants were randomly assigned to receive either a high-nitrate (spinach; 845 mg nitrate/day) or low-nitrate soup (asparagus; 0.6 mg nitrate/day) for 7 days with a 1-week washout period. The results demonstrated that high-nitrate spinach soup administration, containing approximately 845 mg of nitrate, decreased postprandial arterial stiffness measure by 6.93 ± 8.7% (*p* < 0.001 vs. low: −2.28 ± 12.5%, *p* = 0.35) and reduced central systolic blood pressure (cSBP) by 3.39 ± 5.6 mmHg (*p* = 0.004) following 7 days of administration [38]. 

Aatola et al. [39] in a cohort study with 1622 participants found that vegetable consumption during childhood is inversely associated with PWV (β = −0.06, *p* = 0.02) in adulthood. The high consumption of both fruits and vegetables during childhood/adolescence was also associated with lower pulse wave velocity (*p* = 0.03) in adulthood. Furthermore, the Prediction for Atherosclerotic Cardiovascular Disease Risk in China (China-PAR) study, a cohort study in China, included 6628 participants and aimed to assess the association between fruit and vegetable intake and arterial stiffness [40]. The findings of the study showed that high fruit and vegetable intake was associated with a reduced risk of elevated arterial velocity pulse index compared to a lower dose (<500 g/d). The results of the China-PAR project emphasize the importance of adhering to dietary recommendations regarding the daily consumption of the appropriate fruit and vegetable servings [40].

It is important to note that the effects of fruit and vegetable consumption on arterial stiffness can be influenced by overall dietary patterns and lifestyle factors.

### 3.2. Legumes

Like most plant-based foods, legumes, such as peas, lupines, beans, chickpeas, lentils, and soybeans employ a multifaceted approach to confer cardiovascular protection. However, there is much to uncover regarding the related mechanisms, even though the bioactive components of legumes offer valuable insights.

Different varieties/categories of legumes exhibit diverse properties. These variations could be linked to the composition of polysaccharides, namely the amount and variety of starch and fiber in food, the composition of proteins, and the fluctuations in phytochemical content [41,42].

Other bioactive components that are beneficial for vascular health are phytochemicals. These are structurally diverse, naturally occurring compounds that are often found in abundance in fruits, vegetables, legumes, tea, and wine. Polyphenols, flavonoids, isoflavonoids, anthocyanidins, phytoestrogens, terpenoids, carotenoids, limonoids, phytosterols, glucosinolates, phytohaemagglutinins, and fibers are the main categories of phytochemicals [42]. Numerous studies have demonstrated the potent protective effects of some polyphenols against a wide range of clinical diseases, particularly those caused by oxidative stress [43].

According to a review published by Yamagata et al. [44], the dietary intake of different polyphenols improved anti-atherogenic, anti-thrombotic, and anti-hypertensive endothelial functions, although the mechanisms by which these effects are achieved differ depending on the active polyphenols in each food.

Lastly, legumes have a high dietary fiber content and an equal ratio of both soluble and insoluble fibers. A meta-analysis of 10 RCTs reported the beneficial effect of legume consumption on total and LDL cholesterol, compared to the control (Mean difference (MD) = −11.8 mg/dL, 95%CI: 16.1 to −7.5 and MD = −8.0 mg/dL, 95%CI: −11.4 to −4.6, respectively) [45]. A systematic review and meta-analysis of 22 RCTs reported that the consumption of soluble fiber has an overall lowering effect on some risk factors for CVD risk, mainly by reducing systolic (MD = −1.59 mmHg, 95%CI: −2.72 to −0.46) and diastolic blood pressure (MD = −0.39 mmHg, 95%CI: −0.76 to −0.01) at a median intake of 8.7 g of fiber per day (1.45–30 g/day) over a period of up to 7 weeks [46]. This meta-analysis exclusively incorporated RCTs where fiber supplementation was added to the participants’ regular diet, with interventions lasting more than four weeks to adequately assess changes in blood pressure, including both healthy individuals and those with conditions such as hypertension, dyslipidemia, type 2 diabetes, and overweight.

### 3.3. Dairy Products

The current evidence shows contradictory findings regarding the association between dairy product consumption, including milk and dairy products, and the risk of CVD [47,48,49].

The ELSA-Brasil study [50] included 12,892 participants with a mean age of 51.7 ± 8.9 years old. Caucasian subjects and women reported a higher consumption of dairy products. Those participants also reported lower consumption of alcohol and smoking habits, while they were more physically active than participants with a lower consumption of dairy products. Furthermore, they had lower cardiovascular risk factor values, such as blood pressure and glycemia. The higher consumption of dairy products was linked to lower cfPWV (−0.13 m/s) and blood pressure (BP) (−1.3 mmHg), according to the findings of the ELSA-Brasil study [50]. Nevertheless, the researchers concluded that given this study’s limitations, more data would be required to draw definitive conclusions on the association between the consumption of dairy products and blood pressure and arterial stiffness.

A meta-analysis of seven cross-sectional studies involving 16,443 participants did not find any evidence of a significant impact of dairy products on PWV-measured arterial stiffness, specifically. The consumption of cheese (effect size (ES) = −0.04; 95%CI: −0.07 to −0.01) and dairy products overall was weakly associated with lower PWV values (ES = −0.03; 95%CI: −0.04 to −0.01). Yet, the consumption of milk did not appear to have any effect (ES = 0.02; 95%CI: −0.01 to 0.05) [51].

The observed differences may be attributed to various confounding factors, such as the heterogeneity of the included population and the diverse types of dairy products, including variations in fat or sodium content. Additionally, all findings were derived from the systematic reviews and meta-analyses of observational studies, which possess inherent limitations that cannot be entirely excluded, such as residual confounding and reverse causality [47,48,49].

### 3.4. Nuts and Oils

Nuts, along with oils, can play a vital role in vascular health, primarily due to their impact on heart health and inflammation. There is compelling evidence showing that nut intake confers protection against CVD [52]. 

Sarapis et al. [53] examined the effect of high polyphenol olive oil (HPOO) versus low polyphenol olive oil (LPOO) and found no significant differences between the two interventions regarding arterial stiffness, as assessed by AIx and PWV. This study was a crossover study with an intervention duration lasting 3 weeks for each phase with a 2-week washout period between the change of each phase. Similarly, a RCT by Sanders et al. [54] compared the effects of the daily consumption of 0.45, 0.9, and 1.9 g n-3 long-chain polyunsaturated fatty acids (LC-PUFAs) (Eicosapentaenoic acid (EPA): Docosahexaenoic acid (DHA) ratio of 1.51:1) with a placebo (refined olive oil) in a sample of healthy, non-smoking adults (*n* = 312) and found no effect on arterial stiffness. Their study was the first RCT with sufficient statistical power to test this hypothesis. They employed a small dose (3 g) of refined olive oil as a placebo, which has been demonstrated not to affect endothelial function. A standardized protocol was used for the measurement of FMD, requiring participants to avoid high-fat foods, caffeine, or alcohol the day before, as well as strenuous exercise. Measurements were taken after an overnight fast at a consistent time of day by an experienced vascular ultrasonographer. The findings from their study indicate that, in healthy adults without clinically apparent coronary heart disease, n−3 LC-PUFA intakes ranging from 0.45 to 1.8 g/d had no effect on endothelial function.

On the other hand, in a randomized parallel-group study, 60 adults with mild dyslipidemia were randomized to lifestyle modification (LSM) alone or LSM with consumption of 80 g (in-shell) pistachios (equivalent to 40 g or 1.5 oz shelled pistachios) daily for 3 months. The individuals in the pistachio group demonstrated significant improvements in all cardiometabolic parameters. In particular, the consumption of pistachio improved cfPWV, left brachial pulse wave velocity (baPWV), average baPWV, and brachial artery flow-mediated vasodilation (baFMD) (*p* = 0.037, 0.01, 0.07, and 0.046, respectively) [55].

Different meta-analyses have shown a beneficial effect of nut consumption on endothelial function. Neale et al. [56], in their meta-analysis of nine studies (*n* = 652 participants), found that nut consumption led to an improvement in FMD (MD = 0.79%, 95%CI: 0.35 to 1.23, *p* = 0.0004). Another meta-analysis including 10 RCTs also showed an improvement in FMD (MD = 0.41%, 95%CI: 0.18 to 0.63, *p* = 0.001, I^2^ = 39.5%) [57]. However, the beneficial long-term effect was limited only to walnut consumption.

### 3.5. Red Meat, Poultry and Fish

The association between meat consumption and CVD is multifaceted and influenced by several factors, including the type of meat consumed, cooking methods, and overall dietary patterns.

Red meat, such as beef, pork, and lamb, has been linked to an increased risk of cardiovascular disease, especially when consumed in large quantities. This association is hypothesized to be attributed to the saturated fat and cholesterol content in red meat [58]. Processed meats also share this detrimental effect. Processed meats often contain high levels of sodium, nitrate, and other additives, which can be detrimental to heart health and aging [59,60]. The methods used for cooking meat can induce substantial alterations in its chemical composition and physical properties. These modifications can influence both the palatability of the product and the health of consumers [61].

In an observational study including young healthy adults (*n* = 40) aged 18–45 years old, with a mean BMI of 23.6 ± 0.5 kg/m^2^, the consumption of ultra-processed food was positively associated with daytime peripheral pulse pressure (PP) (β = 0.22, 95%CI: 0.03 to 0.41, *p* = 0.027) [62]. On the other hand, unprocessed or minimally processed food consumption was inversely associated with daytime PP (β = −0.27, 95%CI: −0.47 to −0.07, *p* = 0.011), overall and daytime central systolic BP (β = −0.27, 95%CI: −0.51 to −0.02, *p* = 0.035; β = −0.31, 95%CI: −0.58 to −0.04, *p* = 0.024, respectively), and nighttime central PP (β = −0.10, 95%CI: −0.19 to −0.01, *p* = 0.042) [62]. Neither unprocessed nor minimally processed food consumption was associated with AIx nor PWV.

Fish consumption has been shown to improve endothelial function, as determined by peak forearm blood flow, duration of reactive hyperemia, and flow debt repayment (FDR), in a RCT involving postmenopausal women (*n* = 23) with type 2 diabetes mellitus (T2DM) [63]. The patients were randomized to follow a fish-based diet including the consumption of more than 3.0 g/day of n-3 PUFAs or a control diet during a timeframe of 4 weeks in a crossover design. According to the results, the fish-based intervention increased the peak forearm blood flow by 63.7%, the duration of reactive hyperemia by 27.9%, and FDR by 70.7% compared to the control diet.

The Cardiovascular Health Study (CHS) was a multicentered, community-based, prospective study that assessed risk factors for coronary heart disease (CHD) and stroke in older adults [64]. The study included 3931 participants and measured the consumption of animal source food (ASF) and trimethylamine-n-oxide (TMAO)-related metabolites over time. The findings of the study showed that after multivariable adjustment, a higher consumption of unprocessed red meat, total meat, and ASF was related to a higher atherosclerotic cardiovascular disease (ASCVD) risk. This association was also mediated by trimethylamine n-oxide-related metabolites. Contrarily, fish consumption and poultry were not associated with a higher risk of ASCVD. The increase in TMAO concentrations increases oxidative stress, which in turn decreases nitric oxide concentration and endothelial function, leading to increased blood pressure and arterial stiffness [65].

### 3.6. Coffee

The impact of coffee consumption on vascular health has been the focal point of extensive research for many years.

Researchers have identified associations between coffee consumption and endothelial dysfunction, aortic stiffness, and wave reflections. According to a systematic review and meta-analysis, coffee consumption has short-term (postprandial) benefits on endothelial function, as assessed by FMD (MD = 1.93%, 95%CI: 1.10 to 2.75; I^2^ = 97.9%) [66]. However, no significant long-term effect was found (MD = −0.08%, 95%CI: −3.82 to 3.66; I^2^ = 61.4%). While no difference was observed regarding long-term coffee intake, this result should be interpreted with caution due to the limited number of studies and the high risk of bias in the included studies. Further long-term studies with larger sample sizes are necessary to validate our findings.

A cross-sectional study that evaluated the effect of chronic coffee consumption on aortic stiffness and wave reflections in 228 hypertensive patients found that chronic coffee consumption (>450 mL/d) is associated with increased wave reflections, PWV was on average 13% higher, AIx was 2-fold higher, and augmented pressure (AP) was 2.4-fold higher (*p* < 0.01 for all) [67]. Uemura et al. [68] investigated the effects of coffee and green tea consumption on arterial stiffness and found that coffee consumption was significantly inversely associated with ba-PWV, while tea consumption had no effects.

A RCT consisting of 18 participants with overweight or obesity investigated the effects of caffeinated and decaffeinated coffee on the endothelium [69]. According to the results, caffeinated coffee increased central systolic blood pressure (114.06 ± 2.37 vs. 121.94 ± 3.46 mmHg, *p* < 0.001), central diastolic blood pressure (87.77 ± 1.95 vs. 89.88 ± 2.43 mmHg; *p* < 0.001), and PWV (6.03 ± 0.23 vs. 6.31 ± 0.27 m/s, *p* < 0.001), contrary to the decaffeinated coffee. Additionally, there was a better effect on FMD in the caffeinated coffee intake group (*p* = 0.014). 

In healthy individuals, Noguchi et al. [70] found that even a small dose of coffee (54.5 mg caffeine) improves microvascular endothelial function. On the contrary, one RCT showed that caffeine had an unfavorable effect on endothelial function, as assessed by the brachial artery vasoreactivity, in a sample of 20 healthy non-obese subjects [71].

An observational study including 1095 participants found that habitual coffee consumption shows a beneficial relationship with arterial stiffness and blood pressure [72]. More specifically, individuals who consumed coffee and caffeine in small quantities experienced a reduction in PWV (β = −0.15, 95%CI: −0.26 to −0.04), similarly to those who consumed moderate amounts of coffee and caffeine (β = −0.11, 95%CI: −0.19 to −0.02). Additionally, a reduction in peripheral systolic blood pressure was observed among individuals who consumed light and moderate amounts of coffee (β = −3.61, 95%CI: −5.91 to −1.32; β = −2.80, 95%CI: −4.75 to −0.84, respectively). Finally, an inverse association was noted for diastolic blood pressure, both among light (β = −2.48, 95%CI: −4.16 to −0.80) and moderate coffee and caffeine consumers (β = −1.68, 95%CI: −3.11 to −0.26). On the contrary, AIx was not associated with either coffee and caffeine light or moderate drinkers (β = −0.81, 95%CI: −1.27 to 0.36; β = −0.45, 95%CI: −1.27 to 0.15, respectively) [72].

Both coffee and caffeine consumption enhance or impair endothelial function by influencing the balance between vasodilation and vasoconstriction, anti-thrombosis and pro-thrombosis, and anti-inflammation and pro-inflammation, as well as anti-oxidation and pro-oxidation. Caffeine’s impact on endothelial function is more intricate because it acts as both a stimulator and inhibitor of NO, and it also inhibits the degradation of NO’s second messenger, cyclic guanosine monophosphate (cGMP) [73].

Despite many years of research, the immediate and long-term effects of caffeine consumption on arterial wall properties and blood pressure are not yet fully understood. It has been consistently observed that caffeine causes an acute pressor effect, resulting in postprandial increases in aortic pressures and PWV [74].

### 3.7. Alcohol

Alcohol intake has adverse effects on cardiovascular health, leading to reduced myocardial contractility, arrhythmias, dilated cardiomyopathy, and, as a result, the development of progressive cardiovascular dysfunction and structural damage. Whether consumed in binge doses or accumulated over a lifetime, high alcohol consumption is detrimental to the cardiovascular system and should be discouraged. It significantly raises the risk of various cardiovascular complications, including increased rates of overall and cardiovascular mortality, coronary and peripheral artery disease, heart failure, stroke, hypertension, dyslipidemia, and diabetes mellitus.

There is evidence that moderate alcohol consumption is associated with a reduced risk of mortality from cardiovascular disease [75]. However, data suggests that excessive alcohol consumption or a binge drinking pattern is associated with increased arterial stiffness [76]. Del Giorno et al. [77] provided evidence supporting the hypothesis of a threshold effect of alcohol on cardiovascular health. Their findings indicated that low to moderate alcohol intake is associated with lower-than-expected arterial stiffness, while higher doses correlate with accelerated arterial aging. This study aligns with recommendations advocating moderate alcohol consumption, suggesting that both men and women should consume less than 100 g of alcohol per week (i.e., fewer than seven drinks) to maintain cardiovascular benefits. However, several limitations of the referenced study must be acknowledged. The analyzed studies are heterogeneous, and methodological differences need consideration. Although all PWV assessment methods used have been validated, potential measurement biases should be recognized. The majority of studies utilized a cross-sectional design, which does not support causal inference. Various statistical methods were employed to examine the relationship between alcohol consumption and PWV, with most studies relying on correlation analyses and some using multivariate regression analyses with one or more covariates. Notably, the World Health Organization (WHO) released a statement in January 2023 indicating that there is no safe level of alcohol consumption [78].

In a cross-sectional study with 501 participants, it was suggested that heavy alcohol consumption (>70 g/week) is inversely associated with vascular structure, as assessed by carotid intima-media thickness (c-IMT; *p* = 0.019 and arterial stiffness (cfPWV; *p* = 0.039), compared with the consumption of <30 g/week [79]. These results are in agreement with another cross-sectional study (*n* = 3893 participants) that showed that heavy drinkers (≥60 g/day) have a significantly higher PWV compared to moderate (20–39 g/day) and non-drinkers, which in turn correlates with a reduction in vascular stiffness [80].

Despite growing evidence suggesting the potential benefits from moderate consumption of red wine as a therapeutic option to prevent or even treat coronary disease, no solid conclusion has been drawn as it remains uncertain whether the positive effects are influenced by socioeconomic confounders (e.g., age, gender, smoking habits, drinking patterns, physical activity). Additionally, the cardio-protective effects appear to be more pronounced among middle-aged and elderly adults than among young adults [81,82].

Potential confounding factors should be acknowledged to provide a more nuanced understanding of the findings. According to the findings of a systematic review of 19 international longitudinal studies on childhood socioeconomic status (SES) and adult alcohol consumption, there were only weak and inconsistent correlations between childhood SES and alcohol use later in life [83]. Furthermore, a systematic review of five studies indicated that unemployment was linked to a higher level of alcohol consumption. However, it is important to note that the review included a relatively small number of studies, and most of them focused on adolescents. Homelessness can be considered an extreme form of socioeconomic disadvantage and marginalization. For instance, alcohol use among homeless individuals is estimated to be as high as 80 percent, significantly higher than in the general population. A meta-analysis of international studies found an average alcohol dependence rate of 38 percent among the homeless, which is ten times the prevalence of alcohol dependence in the general U.S. population [84].

All the above findings are summarized in Table 1.

## 4. Nutraceuticals

Nutraceuticals, a term derived from “nutrition” and “pharmaceuticals”, refer to bioactive compounds from various natural sources, including foods and dietary supplements that hold potential benefits in treating and preventing various diseases [89]. Given that vascular aging, a significant determinant of cardiovascular and overall health in the elderly, is recognized as a modifiable risk factor, dietary interventions could be considered as a measure of endothelial function maintenance [10]. 

### 4.1. Nitrate and Nitric Oxide (NO)

Nitrate (NO_3_^−^) is an inorganic molecule found in green leafy vegetables, beetroot, and fruits [10,89]. This molecule may play an important role in vascular aging, as its chemical reduction results in the production of nitrite (NO_2_^−^), which is further converted to nitric oxide (NO). NO is involved in the regulation of various vascular aging-related processes, including vascular reactivity regulation, prevention of leukocyte adhesion to endothelial surfaces, and vascular tone and blood flow maintenance, among many others [90,91,92].

NO is produced through two metabolic pathways: NO synthase (NOS) enzymes from l-arginine, and nitrate reduction. Numerous clinical trials have investigated the effects of nitrate on vascular aging-related biomarkers. Webb and colleagues [90] conducted a three-phase crossover clinical trial investigating the acute effects of 500 mL beetroot juice on blood pressure, enterosalivary circulation, and FMD. The authors reported that the consumption of dietary nitrate in the form of beetroot juice resulted in a decrease in BP, with the most significant drop (a drop of 10.4 ± 3.0 mmHg) being observed in systolic BP (SBP), 2.5 to 3.0 h after ingestion (*p* < 0.01). Furthermore, a reduction of 6 mmHg in SBP was reported, 24 h after the ingestion (*p* < 0.01). This was attributed to the fact that nitrate was converted into nitrite by bacteria on the tongue’s surface, which in turn inhibited platelet aggregation. However, no correlation was found between plasma nitrate concentration changes and systolic BP (Pearson r = −0.17, *p* > 0.05). Finally, it was shown that beetroot juice had a protective effect on endothelial function, as shown by a reduction in FMD response in ischemia-reperfusion (I/R) (*p* < 0.05). 

A RCT with a crossover design in 15 healthy older males also supported that beetroot juice consumption improved FMD compared to the placebo (1.18 ± 0.94% vs. 0.23 ± 1.13%, *p* = 0.002) [93]. This research team additionally evaluated biomarkers such as PWV, passive leg movement hyperemia, central pulse pressure, and central systolic pressure, but no changes were observed. However, BP remained stable with either nitrate or placebo supplementation. No effects on BP were also reported in a randomized placebo-controlled double-blind crossover study that included the simultaneous administration of inorganic nitrate and oral glucose in 33 healthy adults, revealing a possible interaction between NO_3_^−^ and glucose levels [94,95]. Similarly, other markers of arterial stiffness, including PWV, cSBP, and AIx, did not differ between the NO_3_^−^ and placebo groups. 

Another RCT with a crossover design in men with overweight and obesity revealed that consuming beetroot juice alongside a meal led to a reduction of the postprandial FMD decrease compared with the control drink (−0.37 ± 2.92% versus −1.56 ± 2.90%, *p* = 0.03) [95]. Similarly, a pilot RCT with a crossover design that used the consumption of a nitrate-rich salad for 10 days as a dietary intervention in postmenopausal women reported that high-nitrate salad consumption led to an increase in FMD compared to the control group (+17% versus −8% respectively; *p* = 0.047, ES = 0.407), but did not affect BP, which might be attributed to the short duration of the 10-day intervention and the generally healthy baseline levels of peripheral blood pressure (PBP) among the participants [96].

In a randomized double-blind crossover study that included 18 drug-naive patients with hypertension, the researchers investigated the effects of a single-dose dietary nitrate in the form of beetroot juice (500 mg/8.1 mmol NO_3_^−^) compared to nitrate-depleted beetroot juice (<0.08 mmol NO_3_^−^), with a washout period of 7–10 days between visits, on arterial stiffness by evaluating the cfPWV. The findings of this study showed that there was no difference between cfPWV in the two groups [97]. 

Furthermore, one RCT that investigated the combined effects of dietary nitrate and pharmacological treatment, and more specifically a 6-month intervention with dietary nitrate (nitrate-containing beetroot juice) and spironolactone or doxazosin, showed no effect on carotid local stiffness but showed a significant reduction on carotid intima-media thickness compared to placebo juice (−0.06, 95%CI: −0.12 to −0.01, *p* =  0.034) [98]. 

Regarding a possible dose-response or treatment duration outcome relationship, one RCT revealed a dose-dependent decrease in blood pressure and vasoprotection after ingestion of inorganic nitrate in the form of either dietary nitrate or supplementation [99]. According to a systematic review and meta-analysis that included 43 RCTs with 765 adults, supplementation of beetroot inorganic nitrate decreased arterial stiffness as measured by PWV (MD = −0.27, 95%CI: −0.51 to −0.02, *p* = 0.04) and improved endothelial function by increasing FMD (MD = 0.627, 95%CI: 0.231 to 1.023, *p* = 0.002), with the dose of inorganic nitrate varying between 70 and 1500 mg/day administered in the form of food or beverages (juice, gel, bread, bars) and the duration ranging between 1 h and 6 weeks [100]. The findings of the subgroup analyses showed that beetroot inorganic nitrate supplementation had no effect on PWV based on the mean age of participants (≥65 vs. <65), the nitrate dosage (<400 mg vs. ≥400 mg), and health status (healthy vs. hypertension). The same findings also apply to the AIx outcome. Contrarily, beetroot inorganic nitrate supplementation had an effect in age ≥65 years participants with hypertension, and with <400 mg of nitrate dosage [100].

Lastly, a RCT included 81 participants with stable chronic obstructive pulmonary disease (COPD) and assigned them to two groups, one receiving 70 mL nitrate-rich beetroot juice (NR-BRJ) and the other receiving identical nitrate-depleted placebo for 12 weeks [101]. The outcomes of the RCT were changes in home SBP measurement, changes in 6 min walking distance (6MWD), and the assessment of the endothelial function. The findings of the RCT demonstrated that compared to the placebo, the intervention group ameliorated 6MWD, reactive hyperemia index (RHI), and AIx75. As a result, subjects with COPD may present a prolonged reduction in BP, which is associated with the amelioration of endothelial function and exercise capacity.

NO bioavailability seems to be lower with aging, especially in sedentary people [92]. Strategies to enhance nitric oxide (NO) bioavailability include the consumption of a diet rich in nitrate-containing foods like leafy greens and beets or nitrate supplements. Regular physical activity, particularly aerobic exercise, may also preserve or increase NO bioavailability in older adults [92,102] Additionally, amino acid supplements like L-arginine and L-citrulline may increase NO levels because they possess specific roles in NO production pathways [103]. Finally, lifestyle interventions including avoiding or reducing behaviors that increase oxidative stress, such as smoking and excessive alcohol consumption, can help maintain NO levels [92].

However, it should be noted that there are potential side effects or risks of high nitrate intake that should be discussed. The lethal dose of nitrate ranges from 4 to 50 g [104], equivalent to 67 to 833 mg per kg of body weight [105]. For nitrite, the lethal dose is set between 1.6 and 9.5 g, corresponding to 33 to 250 mg per kg of body weight. Vulnerable populations such as children, the elderly, and individuals with deficiencies in methemoglobin reductase or cytochrome b5 have been observed to experience adverse effects at lower concentrations [105]. Methemoglobinemia represents the most significant health risk associated with human exposure to nitrates or nitrites [106]. Individuals particularly susceptible to methemoglobin formation include fetuses, infants under 6 months old, and those with genetic defects affecting methemoglobin reductase or nicotinamide adenine dinucleotide (NADH) cytochrome b5.

Certain research findings have raised alarms regarding the potential carcinogenic effects of nitrates and nitrites, substances utilized in meats as preservatives and enhancers of color. Nitrates have the capacity to combine with amino acids, forming nitrosamines, which have been linked to cancer in animal studies. Studies have indicated an increased risk of non-Hodgkin’s lymphoma and cancers affecting the esophagus, nasopharynx, bladder, colon, prostate, and thyroid [107].

Additionally, there is compelling evidence linking the ingestion of nitrate-contaminated drinking water to adverse health effects, beyond methemoglobinemia, including associations with colorectal cancer, thyroid disease, and neural tube defects [108].

It is important to recognize that random within-subject variation could account for much of the individual variability in response to NO_3−_ supplementation. Similarly, determining whether an individual consistently responds or does not respond to NO_3−_ on different occasions may present challenges [109]. However, several factors have been identified that could explain the true differences in response to NO_3−_ among individuals. These factors include age, health status, exercise training status, sex, genetic factors, and variations in the oral microbiome. For example, a RCT assessing whether inorganic nitrate supplementation reduces blood pressure in humans showed that women may benefit more from nitrate supplements compared to men.

More RCTs on the effects of dietary nitrate on different vascular aging-related parameters are necessary, and ongoing research is investigating the impact of dietary nitrate on endothelial function, arterial stiffness, and other related biomarkers [110].

All of the above information is summarized in Table 2. 

### 4.2. Resveratrol

Resveratrol is a natural phenolic compound found in various plant foods such as grapes, peanuts, blueberries, cucumber, tomato, red cabbage, and spinach. This compound can also act as a phytoestrogen and is known for its biofunctional properties, including antioxidant and anti-inflammatory effects, as well as for its potential impact on vascular aging [111,112]. Phytoestrogens belong to the family of phytochemicals, a compound group, which are found in a lot of foods such as soy, vegetables, and fruits, and can be categorized into four principal groups: isoflavonoids, flavonoids, stilbenes, and lignans [111]. 

Regarding the health benefits of resveratrol, in vitro, animal, and human studies have shown that resveratrol is a potential antioxidant nutrient that can reduce oxidative stress, enhance nitric oxide production, and improve antioxidant mechanisms, which in turn may contribute to the protection of organisms against aging-associated vascular diseases [113,114]. The antioxidant activity of resveratrol could be attributed to its chemical structure [111].

Experimental studies indicate that resveratrol (RSV) modulates several processes involved in endothelial dysfunction, including impaired vasorelaxation, endothelial nitric oxide synthase (eNOS) uncoupling, leukocyte adhesion, endothelial senescence, and endothelial–mesenchymal transition [115]. The protective effects of RSV on the endothelium are mediated through various molecular targets, such as Silent Information Regulator 1 (SIRT1), 5′AMP-activated protein kinase (AMPK), eNOS, nuclear factor-erythroid 2-related factor-2 (Nrf2), peroxisome proliferator-activated receptor (PPAR), Krüppel-like factor-2 (KLF2), and nuclear factor kappa-light-chain-enhancer of activated B cells (NF-kB) [115].

One RCT involving 28 otherwise healthy individuals with obesity showed that an intervention with resveratrol supplementation (75 mg) in the form of capsules daily for six weeks led to a 35% greater response in FMD compared to the placebo (*p* = 0.021), but BP remained unaffected [116]. On the contrary, no effects on FMD were found after resveratrol supplementation for 30 days on 48 healthy adults compared to energy restriction [117]. Another clinical trial involving 19 adults who had undergone post-coronary artery bypass graft reported that the effects of resveratrol supplementation (330 mg, 3 × day^−1^) depend on the revascularization method undergone by the patient [118]. 

Finally, a dose-response relationship between resveratrol supplementation and FMD response (*p* < 0.01), which increased from 4.1 ± 0.8% (placebo) to 7.7 ± 1.5% after 270 mg resveratrol, was proposed by a study that included 19 individuals with overweight/obesity and untreated hypertension [119].

Based on the published literature, at present, there are no established consensus treatment regimens for any specific condition or endpoint, except that resveratrol is generally well tolerated at doses up to 1 g per day [120].

In humans, the oral absorption rate of resveratrol is approximately 75%, primarily occurring through transepithelial diffusion. However, extensive metabolism in the intestine and liver reduces its oral bioavailability to significantly less than 1% [121]. Increasing the dosage and administering repeated doses of resveratrol does not seem to significantly affect this bioavailability. To improve the bioavailability of resveratrol, a logical approach has been to develop derivatives. This includes both synthetic derivatives and the evaluation of naturally occurring derivatives, which are sometimes quite abundant in the human diet [121]. 

### 4.3. Flavonoids

Flavonoids are a group of plant compounds known as phytonutrients. There are many different types of flavonoids, including quercetin, catechins, and anthocyanins [122]. There is evidence highlighting that dietary flavonoids and nitrate found in fruits and vegetables may explain the cardioprotective effects of a plant-based diet [123] and may be involved in pathways related to vascular aging [124]. Flavonoids present in almond skin significantly contribute to health benefits and work synergistically with vitamins C and E to protect against LDL oxidation [125]. Additionally, apples are abundant in flavonoids, and green leafy vegetables are rich in dietary nitrate. The research indicates that combining flavonoids with dietary nitrate can enhance nitrous oxide production. This increase in nitrous oxide, resulting from the consumption of flavonoids and dietary nitrate, may improve cognitive function and mood [125].

According to a meta-analysis of 113 RCTs examining the effect of flavonoids on cardiovascular disease risk factors, the intake of flavonoid-rich foods, such as chocolate, improved FMD after acute and chronic intake (MD = 3.99%, 95%CI: 2.85 to 5.12, six studies; MD = 1.45%, 95%CI: 0.62 to 2.28, two studies, respectively) [126]. Cocoa flavanols have also been reported to improve FMD, BP, and vascular stiffness in healthy subjects [127,128,129], but the evidence is controversial. One RCT of parallel design involved 140 postmenopausal women and aimed to assess the impact of the daily consumption of 10 g of high-cocoa chocolate for six months on vascular health. The findings showed no differences in SBP (MD = −1.45 mmHg, 95% CI: −4.79 to 1.88, *p* = 0.391) or baPWV (MD = 0.18 m/s, 95%CI: −0.14 to 0.50, *p* = 0.263) between the intervention and the control group [130].

Furthermore, a flavonoid called proanthocyanidin, and also other polyphenols found in grapes, seems promising in maintaining vascular elasticity and normal blood pressure levels in different population groups, including prehypertensive individuals [131], men with metabolic syndrome [132], mildly hypertensive individuals [133,134], and healthy smokers [135].

At present, there is no consensus on which class of polyphenols is most effective in enhancing vascular health. Nonetheless, a systematic review and meta-analysis suggest that different polyphenol classes exhibit varying levels of effectiveness for the risk of CVD. For instance, proanthocyanins and anthocyanins are more effective in reducing the risk of CHD, whereas catechins are more effective in lowering the risk of stroke [136]. Furthermore, controlled intervention trials have demonstrated that both the chronic and acute consumption of tea improves endothelial function [137,138].

The chronic effects of flavonoid supplementation appear more promising, with minimal conflicting evidence, even when considering the dosage administered. It suggests that a higher dosage may be necessary to produce an immediate effect in acute studies [139]. This could be due to varying absorption rates of flavonoids after ingestion or because the immediate effects are not as pronounced as those resulting from chronic supplementation. However, further research is needed to confirm these theories. Overall, existing evidence indicates that flavanols can enhance peripheral blood flow by increasing NO levels in the endothelium, provided that an effective dose is administered [139].

#### 4.3.1. Quercetin

Quercetin is a flavonoid compound derived from the amino acid phenylalanine known for its antioxidant, anti-inflammatory, and anti-carcinogenic properties, among others [140,141]. The antioxidant activity of quercetin is linked to its ability to scavenge free radicals, including reactive oxygen species (ROS) and reactive nitrogen species (RNS). Just like resveratrol, this effect is attributed to its chemical structure [141].

According to a systematic review and meta-analysis including 17 RCTs with 886 participants, quercetin reduced BP and triglycerides concentration [142]. Furthermore, the consumption of quercetin for periods longer than 8 weeks in the RCTs with a parallel design significantly increased levels of high-density lipoprotein cholesterol (HDL-C).

Another systematic review and meta-analysis of seven RCTs also reported the possible anti-hypertensive properties of quercetin (both in systolic BP (WMD = −3.04 mmHg, 95% CI: −5.75 to −0.33, *p* = 0.028) and diastolic BP (WMD = −2.63 mmHg, 95%CI: −3.26 to −2.01, *p* < 0.001)), particularly in dosages of >500 mg/day [143].

Limited clinical trials have explored the effects of quercetin on vascular aging parameters. One RCT with 72 healthy individuals with overweight or obesity showed that onion peel extract, which is abundant in quercetin, led to an increase in FMD at 12 weeks (from 12.5 ± 5.2 to 15.2 ± 6.1; *p* = 0.002 versus no difference in the placebo group) and circulating endothelial progenitor cells (44.2 ± 25.6 versus 52.3 ± 18.6; *p* = 0.005) compared to the placebo [144]. Furthermore, another RCT including 88 post-myocardial infarction (MI) patients who had experienced their first MI with an age range between 30 and 65 years showed that daily supplementation with quercetin for 8 weeks was not superior to the placebo in improving endothelial dysfunction biomarkers [145].

Finally, it should be noted that quercetin’s effects depend on its concentration. At lower concentrations, it acts mainly as an antioxidant, while at higher concentrations, it may induce oxidative stress, an effect previously called the “quercetin paradox” [140,146].

Quercetin is considered generally safe for consumption as food, recognized as GRAS (Generally Recognized as Safe) [147]. Oral supplementation at doses up to 1000 mg per day for up to 12 weeks has shown no signs of toxicity. However, there is a lack of long-term safety data at high doses, and concerns regarding its potential carcinogenic effects remain unresolved. High doses exceeding 1000 mg per day have been linked to kidney damage. Animal studies indicate a possible risk of promoting tumors in estrogen-dependent cancers. Additionally, there is concern that a quercetin byproduct may interfere with protein function. Quercetin has the potential to interact with medications, such as pravastatin, fexofenadine, and cyclosporine, leading to altered drug bioavailability [148]. It is advisable to avoid combining quercetin with the cardiac glycoside digoxin [149]. There are available studies indicating that there is supplement–drug interaction of quercetin with tamsulosin on vasorelaxation, as well as with CYP2C9 enzyme activity of diclofenac [150]. There are also two published case reports showing that quercetin may interact with anticoagulants by increasing their effect [151,152].

Previous animal and human studies have demonstrated that quercetin exhibits poor oral bioavailability after a single dose, primarily due to macronutrient absorption. As a lipophilic compound, quercetin is believed to cross intestinal membranes via simple diffusion, potentially offering better absorption compared to its glycoside forms, which reach the intestines without degradation [153].

Quercetin has been found to competitively bind to bacterial DNA gyrase, making it contraindicated for use with fluoroquinolone antibiotics. Additionally, quercetin is a potent competitive inhibitor of CYP3A4, the enzyme responsible for drug metabolism in the body, and is thus predicted to increase serum concentrations of drugs metabolized by this enzyme, such as diltiazem [153].

#### 4.3.2. Tea Catechins

Catechins are flavonols found in various plant-based foods and beverages, including green tea. Catechins are considered to possess direct and indirect antioxidant properties. These compounds scavenge ROS and remove heavy metals involved in the generation of free radicals, a process known as metal ion chelation [154].

The indirect antioxidant mechanisms of catechins include the induction of antioxidant enzymes, the inhibition of pro-oxidant enzymes, and the suppression of oxidative stress and inflammation-related pathways [154]. Numerous age-related conditions, such as neurodegenerative diseases, cardiovascular diseases including endothelial dysfunction or hypertension, and diabetes, have been linked to oxidative stress and inflammation [155]. Therefore, catechins are a potent dietary countermeasure for these conditions [89]. Epidemiological studies have reported a favorable relationship between tea consumption and cardiovascular health [156,157].

A recent hypothesis suggests that the beneficial effects of green tea on coronary artery disease may be due to its preventive effects on chronic inflammatory diseases. Considering the current focus on the inflammatory nature of coronary artery disease, green tea’s anti-inflammatory properties could explain the paradoxical finding that green tea did not protect against coronary artery disease in Japanese patients but did protect against myocardial infarction [158].

Extensive evidence substantiates the manifold cardioprotective benefits associated with consuming catechins, including lowered systemic blood pressure, enhanced FMD, and mitigation of atherosclerosis, platelet activation, and thrombosis formation. Nonetheless, the translation of statistically significant findings from trials into clinically meaningful reductions in long-term cardiovascular outcomes remains uncertain [159]. 

Regarding the effect of catechins on the specific markers of endothelial function and arterial stiffness, according to a crossover trial involving healthy individuals, consuming both green and black teas resulted in improvements in baFMD. The baseline FMD, which started at approximately 5.4% and 5.0% for green and black tea consumption, respectively, increased to around 10.2% and 9.1% two hours after drinking green and black tea, respectively (*p* < 0.001) [160]. Another RCT in a crossover design reported a dose-dependent relationship between tea consumption and endothelial function in healthy men. Black tea demonstrated a dose-dependent effect on FMD, leading to an increase from 7.8% in the control group to 9.0%, 9.1%, 9.6%, and 10.3% in response to varying flavonoid doses (100, 200, 400, and 800, respectively) (*p* = 0.0001). Notably, even the lowest investigated daily dose of 100 mg (which equals to <1 cup of tea) improved FMD by 1.2% compared to the control group (*p* = 0.0113) [161]. FMD improvement after 800 mg/day was significant compared to the control (*p* < 0.0001), but so were 100 mg/day (*p* = 0.0121) and 200 mg/day (*p* = 0.0275).

However, there was no difference between the consumption of different teas and control groups in the cfPWV (MD = −0.19 ± 0.5 m/s, *p* = 0.903). Several trials in the literature report a favorable effect of green and black tea in vascular aging, as assessed by baFMD [137,162,163,164]. However, the results should be carefully interpreted, as there is a notable heterogeneity in the population and the duration of the interventions [91]. 

The only systematic review and meta-analysis in the literature that explores the effects of catechins on vascular aging reported that catechin supplementation led to an increase in FMD (MD = 1.53, 95%CI: 0.93 to 2.14) and reduced PWV (MD = −0.32, 95%CI: −0.44 to −0.20) and AIx (MD = −3.57, 95%CI: −6.40 to −0.74), but did not affect other markers of endothelial function, such as intercellular adhesion molecule-1 (ICAM-1) and vascular adhesion molecule-1 (VCAM-1) [165]. According to the findings of a subgroup analysis, it was shown that FMD was improved regardless of the age of participants (≥45 vs. <45) and the health status of participants (healthy vs. heart disease) [165]. The same findings also apply to the PWV outcome regarding participants’ age.

The limitations of the included studies are noteworthy, as they involve diverse populations; some studies included healthy participants, while others focused on individuals with CVD. Additionally, the studies varied in their assessment of catechin effects, with some examining acute effects and others chronic effects. There were also differences in the interventions regarding catechin supplementation, such as the consumption of green or black tea. Furthermore, one study used water as the control group, which hindered the possibility of double-blind masking. Previous research indicates that creating a placebo beverage that mimics the taste of tea without containing tea flavonols is unfeasible. Moreover, the participants’ coffee consumption could potentially influence the long-term findings of the cited studies, as coffee appears to have no short-term effect.

Based on the currently available evidence, no recommendation can be made regarding the optimal level of tea consumption. Since safety issues are concerned, it has been stated in a scientific opinion from the European Food Safety Authority (EFSA) that catechins found in green tea developed in a traditional way and in reconstituted drinks, with similar compositions, are generally considered safe, as long as the intake levels align with those reported in European Member States [166]. The evidence from interventional clinical trials has shown that an intake of doses equal to or greater than 800 mg epigallocatechin-3-gallate (EGCG)/day taken as a dietary supplement causes a significant increase in serum transaminases in treated subjects compared to the control. Catechins present in green tea extracts, whether consumed as a beverage or in liquid or dry form as dietary supplements, may exhibit higher concentrations and differ in composition and consumption patterns compared to catechins from traditional green tea infusions. Consequently, they cannot be considered safe under the presumption of a safety approach [166].

Tea is classified into three types based on fermentation levels: green (unfermented), oolong (partially fermented), and black (fermented). Generally, green tea extracts exhibit stronger antioxidant activity than semi-fermented and black tea extracts, primarily due to their higher content of (−) epigallocatechin gallate. The manufacturing process of black tea significantly reduces the levels of monomeric catechins compared to the less intense processing of other teas [167].

#### 4.3.3. Anthocyanins

Antioxidants are compounds that protect the body’s cells from oxidative stress. Dietary antioxidant intake has been linked to an improvement in vascular aging-related parameters, such as carotid intima-media thickness [168] and higher levels of plasma antioxidants may be protective against early atherosclerosis. According to a crossover study in 14 healthy adults who consumed 200 g of cooked purple potato containing anthocyanins or a white potato without anthocyanins for 14 days, PWV was decreased after the consumption of the purple potato variety (−0.3 ± 0.5 m/s vs. + 0.1 ± 0.6 m/s) [169]. Anthocyanins have been reported to improve FMD after acute (SMD: 3.92%, 95%CI: 1.47 to 6.38) and chronic (SMD = 0.84%, 95%CI: 0.55 to 1.12) administration compared to the control group. On the other hand, only chronic supplementation with anthocyanins led to an improvement in the PWV (SMD = −1.27 m/s, 95%CI: −1.96 to −0.58) [170]. However, a recently published RCT showed that there was no difference in FMD between the group assigned to anthocyanin supplementation and the control group [171].

### 4.4. Coenzyme Q10

Coenzyme Q10 (CoQ10) is a vitamin-like benzoquinone that acts as an electron carrier in the mitochondrial respiratory chain and also functions as an essential intracellular antioxidant [172,173]. Coenzyme Q10 is found in two forms: ubiquinone, which is the oxidized form, and ubiquinol, which is the reduced form. Coenzyme Q10 is known for its lipophilic antioxidant properties, and specifically for effectively countering free radicals and regenerating reduced vitamin E. Various factors, such as genetics, aging, and statin treatment can lower CoQ10 concentration and result in CoQ10 deficiencies, which are linked to conditions involving oxidative stress, like neurodegenerative disorders, diabetes, cancer, and cardiovascular diseases [172]. CoQ10-rich foods include meat, fish, nuts, and specific oils. 

Impaired baFMD is a common problem in patients undergoing hemodialysis [174]. One observational study in this population reported a significant correlation between FMD and the plasma CoQ10 level (r = 0.727, *p* < 0.001). CoQ10 concentrations were an independent predictor of better FMD, as determined by applying a stepwise multivariate linear regression analysis (β = 0.018 for each 10 ng/mL increase, *p* < 0.001). 

The favorable effects of CoQ10 supplementation were also confirmed by other clinical trials on various population groups [174,175,176]. Finally, according to a systematic review and meta-analysis of five trials and 194 patients, treatment with coenzyme CoQ10 improved endothelial function, as assessed by flow-mediated dilatation (SMD = 1.70, 95%CI: 1.00 to 2.40, *p* < 0.0001) compared to the placebo [177].

CoQ10 shares structural similarities with vitamin K and may counteract the effects of warfarin [178]. However, it has also been reported that CoQ10 may increase the risk of bleeding when taken with warfarin. Additionally, CoQ10 may delay the clearance of theophylline, potentially leading to persistent vomiting, cardiac arrhythmias, and intractable seizures [179]. Furthermore, in a murine model of non-small cell lung cancer, the intake of ubiquinone appeared to mitigate the effects of radiation therapy [180].

CoQ10 has the potential to interact with specific medications, such as anticoagulants, antihypertensives, and chemotherapeutic agents. CoQ10 has been found to reduce the effectiveness of warfarin, a widely used anticoagulant, thereby increasing the risk of thrombosis [181]. Moreover, CoQ10 may interfere with the action of antihypertensive medications. A review indicates that patients with the lowest baseline serum levels of coenzyme Q10 exhibit the most significant antihypertensive response to supplementation. Consequently, these individuals may also experience a more pronounced enhancement in vascular health compared to those with higher initial concentrations of coenzyme Q10 [182].

A pilot study presents evidence suggesting that NQO1P187S and apoE polymorphisms affect CoQ10 status in humans [183].

Overall, evidence from preclinical and clinical studies demonstrates that CoQ10 supplementation exhibits a high safety profile and is well tolerated at doses significantly exceeding typical levels, even over extended durations, with minimal adverse effects [172]. Notably, the administration of doses up to 3000 mg/day in humans has not been associated with serious adverse effects. However, gastrointestinal symptoms may occur when doses exceed 1200 mg/day per individual. It is important to note that the safety of CoQ10 supplementation in children, during pregnancy, and during lactation has not been established.

All of the above information is summarized in Table 3.

### 4.5. Curcumin

Curcumin is a bioactive compound found in turmeric and is often used as a dietary supplement due to its antioxidant and anti-inflammatory properties [184]. According to a RCT that included 39 healthy men and postmenopausal women, curcumin supplementation at a dose of 200 mg per day increased FMD (5.7 ± 0.4 vs. 4.4 ± 0.4% at baseline, *p* = 0.001) but did not affect aortic pulse wave velocity or carotid artery compliance [185]. On the contrary, 500 mg curcumin for 12 weeks improved cfPWV compared to the placebo (−10.9 ± 0.83 vs. −0.43 ± 1.24, *p* = 0.03) in 66 patients with metabolic syndrome in a randomized placebo-controlled trial. Conversely, there was no difference in the two groups regarding aortic AIx and AIx75 (1.12 ± 9.34 vs. −0.46 ± 7.2, *p* = 0.46; 0.43 ± 9.83 vs. −1.17 ± 7.89, *p* = 0.49, respectively) [186].

A systematic review and meta-analysis including 35 RCTs assessed the effects of curcumin supplementation compared to the placebo on vascular health markers [187]. The findings of the aforementioned SR and MA showed that curcumin supplementation improved the FMD but not the PWV. This could be attributed to the fact that FMD is primarily influenced by the function of NO as a key circulatory vasodilator. Given the presumed effect of curcumin on enhancing NO bioavailability, it is reasonable to assume that curcumin supplementation is effective in normalizing FMD. Conversely, the ability of curcumin to improve parameters dependent on the physical properties of the endothelial wall, such as PWV, remains unresolved. Even though this SR and MA could not perform a subgroup analysis for the outcomes of FMD and PWV due to the limited number of included studies, it performed a subgroup analysis for SBP, showing that there is no difference for health status with only one exception, people with NAFLD, curcumin dosage, age, and type of intervention with only one exception, unformulated curcumin. More specifically, among the health status categorization, curcumin supplementation improved SBP in healthy participants, patients with diabetes and dyslipidemia, and patients with MetS and prediabetes. On the other hand, there was no difference for patients with NAFLD. Furthermore, the type of intervention, i.e., turmeric, high absorption curcumin, and nano-curcumin, was found to be superior to the placebo in improving SBP. Contrarily, there was no difference for unformulated curcumin [187].

The primary limitation in the application of curcumin is its low bioavailability. This issue is predominantly attributed to poor gastrointestinal absorption, fast metabolism, and rapid systemic clearance [188]. Consequently, the development of strategies to enhance the bioavailability of curcumin is imperative.

To enhance curcumin’s bioavailability, several strategies can be employed, including the use of adjuvants like piperine, complexed or encapsulated curcumin, specialized curcumin formulations, and curcumin nanoparticles [188]. Piperine, an alkaloid derived from black pepper (*Piper nigrum*), functions as a bio-enhancer by inhibiting the activity of UDP-glucuronosyltransferase, CYP3A4, and P-glycoprotein, thus improving curcumin absorption and metabolic stability. Piperine has been demonstrated to significantly increase the bioavailability of various compounds by inhibiting drug-metabolizing enzymes and enhancing curcumin absorption and systemic availability [188]. Another strategy to enhance curcumin’s bioavailability involves the use of liposomal formulations [189]. Liposomes, which are phospholipid-based vesicular systems, are among the most extensively studied lipid drug carriers. Their effectiveness is attributed to their amphiphilic nature, high biocompatibility, and strong affinity for biological membranes. These characteristics allow for rapid uptake through pinocytosis or facilitated diffusion across lipophilic cell membranes [189].

Curcumin has a well-established safety profile. According to reports from the Joint United Nations and World Health Organization Expert Committee on Food Additives (JECFA) and EFSA, the Allowable Daily Intake (ADI) value for curcumin ranges from 0 to 3 mg/kg body weight [190].

While curcumin is generally considered safe, adverse effects have been reported at higher doses. These can include diarrhea, headache, rash, and yellow stool with doses ranging from 500 to 12,000 mg. Furthermore, excessive daily intake may lead to nausea and increased levels of serum alkaline phosphatase and lactate dehydrogenase [190].

Curcumin has the potential to influence the pharmacokinetics of various conventional drugs. Its interactions have been studied with several classes of medications, including chemotherapeutic agents, antihistamines, analgesics, antidepressants, cardiovascular agents, antibiotics, immunomodulators, and anticoagulants [191]. Notably, curcumin may enhance the effects of antidepressants, such as fluoxetine, when administered concurrently. It can increase the concentration and efficacy of cardiovascular agents like losartan and rosuvastatin while potentially decreasing the absorption and efficacy of talinolol. Additionally, curcumin may enhance the actions of antihistamines, antineoplastic agents, antibiotics, and anticoagulants. However, curcumin may inhibit hemostasis, and its concurrent use with anticoagulants such as warfarin and clopidogrel could increase the risk of hemorrhagic complications, including bruising and bleeding [191].

### 4.6. Omega-3 Fatty Acids

Omega-3 fatty acids are a group of essential polyunsaturated fats that need to be consumed through dietary sources since they cannot be synthesized in the human organism. Several meta-analyses and RCTs have investigated the effects of omega-3 fatty acids on vascular health, with the majority of them concluding on the fact that omega-3 fatty acids consumption leads to improvements in FMD, PWV, and cIMT [192,193,194,195,196,197,198]. More specifically, a systematic review and meta-analysis that included 16 RCTs and 901 participants [198] found that the supplementation with omega-3 fatty acids was superior to the placebo group in increasing FMD (MD = 2.30%, 95%CI: 0.89 to 3.72). On the other hand, it was shown that there was no difference between the two groups regarding endothelium-independent vasodilation. Moreover, the Toon Health study, which included 1803 Japanese adults aged 30–84, demonstrated that omega-3 fatty acid intake was associated with lower odds of severely increased cIMT (OR_adj_ = 0.55, 95%CI: 0.31 to 0.97, p_trend_ = 0.04) [195]. 

However, there are also studies that found no correlation between omega-3 ingestion and the abovementioned parameters [199,200].

A systematic review and meta-analysis regarding the safety of supplementation with omega-3 polyunsaturated fatty acids suggested that subjects receiving omega-3 fatty acid supplements exhibit a higher incidence of minor side effects, including diarrhea, dysgeusia, a tendency to bleed, and general discomfort compared to those receiving a placebo [201].

### 4.7. Astaxanthin

Astaxanthin is a carotenoid produced by microalgae. It is mainly found in seafood, and it exhibits antioxidant and anti-inflammatory properties [202]. Astaxanthin’s role in preserving vascular health has been attributed to the fact that this compound combats oxidative stress, one of the main contributors to vascular aging [203]. A potential mechanism explaining this action is astaxanthin’s ability to neutralize these ROS, thus reducing oxidative stress and its detrimental effects on vascular tissues [204]. Furthermore, astaxanthin’s anti-inflammatory effects play a vital role in vascular aging management, as chronic inflammation can trigger vascular dysfunction and accelerate the aging process [205]. There are very limited randomized controlled studies investigating the effects of astaxanthin in vascular health, with the existing ones combining astaxanthin with medical food [206] or other functional compounds [207] rather than assessing its efficacy as a molecule per se.

### 4.8. Selenium

Selenium is a trace element that plays a crucial role in maintaining vascular health and mitigating the effects of aging thanks to its antioxidant properties [208]. Selenoproteins, including glutathione peroxidases (GPXs), which contain this trace element, function as antioxidants. These enzymes are responsible for neutralizing reactive oxygen species (ROS) and free radicals, thus protecting blood vessels and tissues from oxidative damage. Selenium also possesses anti-inflammatory properties and influences calcium homeostasis, which is a significant factor in vascular function [208]. A 10-year cohort study was conducted to examine the long-term relationship between serum selenium concentrations and various markers of large artery structure and function, including atherosclerosis, arterial stiffness, and blood pressure in adults in South Africa. The results showed that normal selenium concentrations appear to have a vascular protective effect on individuals, which was associated with lower arterial stiffness, as assessed by cfPWV and BP after 10 years. However, in individuals with the highest selenium concentrations, there was a potential detrimental association with cIMT. Therefore, selenium plays a vascular protective role, but excessive levels may have adverse effects [209].

All of the above information is summarized in Table 4.

## 5. Supplements

In recent times, there has been a significant focus on the use of supplementation, resulting in widespread utilization of supplements across the globe [210]. Supplements provide an alternative method to prevent or treat vascular dysfunction for people who often fail to meet the recommendations regarding optimal exercise levels and dietary patterns [16]. Various studies have assessed the effects of different vitamin interventions on vascular health, revealing diverse outcomes [211].

### 5.1. Magnesium

Shechter and colleagues [212] performed a RCT including 50 patients with coronary artery disease with a mean age ± SD of 67 ± 11 in order to assess the effect of oral magnesium intervention compared to the placebo on brachial-artery EF. FMD was defined as the endpoint for evaluating EF in this study. After 6 months of intervention, the participants assigned to the intervention group improved their FMD (15.5 ± 12.0%, *p* = 0.02), while subjects assigned to the control group did not change their FMD from the baseline (4.4 ± 2.5%, *p* = 0.78). Furthermore, at the end of the study, the FMD change (ΔFMD) was greater in the intervention group in comparison with the control group (25.2 ± 13.0% vs. −4.5 ± 2.5%, *p* < 0.02).

Another RCT published in 2016 by Joris and colleagues [213] assessed the effect of long-term magnesium supplementation compared to the placebo on arterial stiffness in subjects with overweight or obesity. Overall, 52 participants were randomized and assigned to the two groups; the one in the intervention group received three times per day a magnesium supplement while the subjects in the control group received placebo capsules. AS was evaluated using the cfPWV. There was a significant difference between the two groups after 24 weeks on cfPWV values (MD = −1.0 m/s, 95%CI: −1.6 to −0,4, *p* < 0.01). Contrarily, there was no change in the central augmentation index adjusted for heart rate (CAIxHR75%) between the two studied groups (MD = 0.2, 95%CI: −2.5 to 2.8).

Finally, Schutten and colleagues [214] conducted a RCT in order to compare the effects of different magnesium formulations, more specifically organic and inorganic magnesium supplements, on arterial stiffness for 24 weeks in subjects with overweight or slight obesity. A total of 164 subjects, the majority of them being women with a mean age ± SD of 63.2 ± 6.8 years, were included in this study. A total of 46 participants were allocated to each intervention group (magnesium citrate, magnesium oxide, and magnesium sulfate), while 26 subjects were allocated to the control group. After 24 weeks of intervention, there was no difference in cfPWV between the intervention groups and the control group (magnesium citrate: MD = 0.0 m/s, 95%CI: −0.7 to 0.6,; magnesium oxide: MD = 0.0 m/s, 95%CI: −0.6 to 0.6; magnesium sulfate: MD = −0.3 m/s, 95%CI: −1.0 to 0.3). The same findings were also evident for the augmentation index adjusted for heart rate (AIxHR75%).

A systematic review and meta-analysis were conducted to assess the effect of oral magnesium supplementation on PWV and FMD as markers of vascular function. The study included five RCTS of a total of 202 participants [215]. The dose of magnesium in the included studies ranged from 323 to 600 mg/day, and the duration ranged from 2 to 6 months. The overall analyses did not result in an association between magnesium supplementation and FMD (MDe = 2.13, 95%CI: −0.56 to 4.82, *p* = 0.12) or PWV (MD = −0.54, 95%CI: −1.45 to 0.36, *p* = 0.24). However, according to the subgroup analysis, magnesium supplementation improved the FMD in the unhealthy population (MD = 3.67, 95%CI: 0.78 to 6.56, *p* < 0.005), participants over 50 years old (MD = 3.67, 95%CI: 0.78 to 6.56, *p* < 0.005), and among those with higher BMI values (≥25 kg/m^2^) (MD = 5.59, 95%CI: 1.01 to 10.17; *p* < 0.005). However, the supplementation of magnesium for over 6 months improved FMD (MD = 3.67, 95%CI: 0.78 to 6.56, *p* < 0.005) compared to the placebo [215]. Contrarily, it was shown that magnesium supplementation had no effect based on sample size, gender, baseline Mg concentrations, and Mg dose ≥350 mg daily.

Magnesium supplementation offers potential long-term benefits for vascular health. Specifically, supplementation with magnesium has been shown to enhance islet beta-cell response and insulin sensitivity in patients receiving thiazide treatment and those with type 2 diabetes [216]. Moreover, continuous oral magnesium supplementation is associated with improved blood pressure control, manifested by reductions in both systolic and diastolic blood pressure, as well as enhanced endothelial function and amelioration of subclinical atherosclerosis [216]. Magnesium supplementation has also been demonstrated to decrease systemic vascular resistance and diminish the incidence of isolated ventricular premature complexes [217].

As far as the safety of magnesium supplementation is concerned, magnesium supplementation has the potential to alter the pharmacokinetics of various medications and increase the risk of adverse effects. This supplementation may interact with drugs such as cellulose sodium phosphate and digoxin [218,219]. Moreover, magnesium can impede the absorption of specific medications or even invert their action, including tetracycline antibiotics (e.g., demeclocycline, doxycycline, minocycline, tetracycline) [220], bisphosphonates (e.g., alendronate) [221], or quinolone antibiotics (e.g., ciprofloxacin, levofloxacin) [220].

### 5.2. Vitamin B

A Japanese study conducted by Maruyama et al. [222] evaluated the effect of vitamin B supplementation on vascular function, as assessed by FMD. The study included 127 participants who had at least one factor of the metabolic syndrome but were not receiving any medication. The intervention was based on a supplement drink containing vitamins B6, B12, and C and folate that participants consumed for 2 months, either at the beginning (early intervention group) or at the end of the study (late intervention group). FMD, serum B vitamins, and vitamin C concentrations were evaluated. No changes were found for %FMD in either the early or later intervention groups.

A double-blind, placebo-controlled, randomized crossover study performed by Kwok and colleagues [223] evaluated the effects of an oral vitamin B12 supplement (500 mg/day) compared with an image-matched placebo on FMD and IMT in vegetarians. BaFMD was found to be increased (6.3 ± 1.8% to 6.9 ± 1.9%; *p* < 0.0001), whereas cIMT was found to be diminished (0.69 ± 0.09 mm to 0.67 ± 0.09 mm; *p* < 0.05) following the B12 supplementation.

### 5.3. Folic Acid

A systematic review and meta-analysis of RCTs evaluated the effectiveness of folic acid supplementation without a vascular challenge on endothelial function, measured by FMD [224]. The analysis included 14 studies with a total of 732 participants. The study arms included intake of folic acid and vitamin B6 or vitamin B12 alone or with both. The median folic acid dose was 5000 μg per day. The vascular reactivity was measured by FMD and %FMD. Out of the fourteen studies, only five studies used vitamin B6 and B12 supplementation as the intervention. Overall, the pooled estimate results showed that the folic acid supplementation improved the %FMD (1.08, 95%CI: 0.57 to 1.59; *p* = 0.0005) compared to the placebo. In the post hoc analysis, the results of FMD showed that a dose-response effect could be detected. The %FMD did not change (−0.07, 95%CI: −0.37 to 0.22) at doses between 400 and 800 μg per day, but for higher doses (10,000 μg/d), the change in %FMD was higher (2.04, 95%CI: 1.43 to 2.65) [224].

Another more recent meta-analysis aimed at investigating the dose-response effect of folic acid and endothelial function [225]. Twenty-one (21) studies were included in this analysis with a total of 1010 cases and 1015 controls (a total of 2025 participants). The endothelial function was measured by %FMD and FMD. The findings showed that folic acid supplementation can increase the %FMD (WMD = 2.59%, 95%CI: 1.51 to 3.67, *p* <  0.001) in all subgroups. Moreover, according to the subgroup analysis, the supplement intake resulted in an increase of FMD levels when the intervention dose was ≥5 mg/day (WMD = 31.05, 95%CI: 5.3 to 56.80, *p* = 0.018) and the intervention performed in the CVD group (WMD = 36.16, 95%CI: 3.35 to 68.98, *p* = 0.031) [225].

More studies on folic acid and arterial stiffness have also been conducted. A recent systematic review and meta-analysis assessed the use of folic acid supplementation and arterial stiffness measured by FMD, PWV, and AIx [226]. Of the total 36 eligible studies, only eight included healthy participants, while the rest of them included patients with various pathological conditions. There were significant differences between folic acid supplementation and the placebo on FMD (SMD = 0.888, 95%CI: 0.447 to 1.329; *p* < 0.001), but no differences on PWV (SMD = −0.069, 95%CI: −0.264 to 0.125, *p* = 0.485) and peripheral PWV (SMD = −0.093, 95%CI: −0.263 to 0.077, *p* = 0.284) were found [226].

### 5.4. Zinc

Zinc has also been examined for its beneficial effect on vascular function. A randomized study conducted by Seet et al. [227] included 40 male participants with type 2 diabetes. The intervention group received zinc supplements (240 mg/day), while the control group received a placebo for a period of 3 months. The vascular function was evaluated by AIx and PWV, but no improvement was reported.

### 5.5. Vitamin C and E

Rasool et al. [228] aimed to evaluate the supplementation of vitamin E and its effect on arterial stiffness through a crossover-controlled study in 20 postmenopausal women. The study participants were randomized to receive either a vitamin E supplement (400 IU) or a placebo. PWV was measured on the fifth week out of the total 10 weeks of the intervention. Following treatment, vitamin E plasma levels were increased, but the association between vitamin E and PWV was not significant and PWV did not differ between vitamin E and the placebo (9.04 ± 0.29 m/s vs. 9.14 ± 0.29, respectively).

Moreover, another study examined the association between vitamin E and endothelial function. The study included 41 participants with type 1 diabetes who were randomly allocated to the intervention group (vitamin E supplement) or the control (placebo) group. The intervention lasted for 3 months and the dose of vitamin E treatment was 1000 IU per day. Regarding endothelial function as evaluated by FMD, there was an improvement following the intake of vitamin E supplements (2.6 ± 0.6% vs. 7.0 ± 0.7%; *p* < 0.0001 for treatment by period interaction effect, *p* < 0.0084 for treatment effect, and *p* < 0.0001 for period effect) [229].

Ashor and colleagues [230] conducted a randomized double-blind crossover trial with the following interventions: (1) NO_3_^−^ supplementation (active) + vitamin C (active); (2) NO_3_^−^ supplementation (active) + vitamin C (placebo); (3) NO_3_^−^ supplementation (placebo) + vitamin C (active); (4) NO_3_^−^ supplementation (placebo) + vitamin C (placebo). The participants were people with a normal or above-normal weight. Vitamin C supplementation resulted in diminished PWV (vitamin C intervention: Δ −0.70 ± 0.31 m/s; vitamin C placebo: Δ 0.43 ± 0.30 m/s; *p* = 0.007) when compared to the placebo. The c combination of vitamin C and nitrate reduced PWV in participants of older age (PWV = 2.0 m/s, *p* = 0.02).

A RCT performed by Mullan et al. [231] included 30 patients with T2DM who were randomized with a 1:1 ratio to either receive an oral ascorbic acid supplementation (500 mg/day) or a matched placebo. AIx% was found to be reduced after the intervention (*p* = 0.026).

A systematic review and meta-analysis conducted by Ashor et al. [230] evaluated 46 RCTs with a total of 1817 participants with respect to the effects between vitamin C and vitamin E supplements or their combination on endothelial function. The analysis that was conducted from studies with vitamin C supplementation alone showed an improvement in endothelial function (SMD = 0.25, 95%CI: 0.02 to 0.49, *p* = 0.043). Moreover, vitamin E supplementation alone also showed an improvement in endothelial function (SMD = 0.48; 95%CI: 0.23 to 0.72; *p* = 0.0001). The combined supplementation of vitamins C and E did not lead to any improvement of endothelial function (SMD = 0.12, 95%CI: −0.18 to 0.42, *p* = 0.428).

### 5.6. Vitamin D

A systematic review and meta-analysis of randomized controlled trials was carried out by Rodriguez and colleagues [232] in order to evaluate the effects of vitamin D supplementation versus the control on PWV and/or AIx in patients with various pathological conditions. Neither PWV nor AIx were affected by the intervention (SMD = −0.1, 95%CI: −0.24 to 0.04; SMD = −0.15, 95%CI: −0.32 to 0.02, respectively). The study design exhibited considerable variability in vitamin D dosing protocols (ranging from 1000 to 5700 IU/day), follow-up durations (ranging from 1 to 12 months), and sample sizes (ranging from 29 to 183 participants). For studies that reported baseline differences in 25-OH vitamin D levels (prior to treatment), no significant differences in mean concentrations were observed between the experimental and control groups. Among studies reporting follow-up vitamin D concentrations, only one study found no differences between the experimental and control groups. Eight studies documented significantly higher 25-OH vitamin D levels in the experimental groups compared to controls. Additionally, only two studies indicated that the experimental groups did not achieve mean vitamin D adequacy (>20 ng/mL) following the intervention.

Witham and colleagues [233] carried out a parallel-group, double-blind, randomized placebo-controlled trial in which healthy South Asian women with a baseline serum 25-hydroxyvitamin D levels of <75 nmol/L were randomized to receive a single dose of 100,000 units of oral vitamin D3 or a matching placebo. According to the results, it was observed that there was no improvement in FMD in the vitamin D group compared to the placebo at 4 weeks (MD = 0.1%, 95%CI: −0.9 to 1.1, *p* = 0.84) or 8 weeks (MD = 0.0%, 95%CI: −1.4 to 1.4, *p* = 0.98). In another RCT by Witham et al. [234], patients who had undergone stroke received ergocalciferol as a single oral dose of 100,000 units of vitamin D2 or an identical placebo. FMD was higher in the intervention group after the timeframe of 8 weeks (mean %change (SD) = 6.9 (3.5) vs. 3.7 (3.1); *p* = 0.007).

Kumar et al. [235] conducted a RCT that aimed to assess the effect of cholecalciferol supplementation compared to the control group on brachial artery FMD in patients with chronic kidney disease (CKD). The FMD showed an improvement in the intervention group (between-group difference in mean change = 5.49%; 95%CI: 4.34 to 6.64), which remained after the adjustment for baseline FMD (*p* < 0.001). Additionally, PWV was also improved (between-group difference in mean change = −1.24 m/s, 95%CI: −2.16 to −0.74).

Another RCT evaluated the effect of long-term high-dose vitamin D supplementation on arterial stiffness parameters as assessed by AIx and PWV [236]. This study was a prespecified analysis of the Vitamin D Assessment (ViDA) study. Briefly, 5110 participants were randomized and allocated to either the vitamin D group (*n* = 2558) or the placebo group (*n* = 2552). After 1 year of follow-up, a total of 517 subjects with an age ranging from 50 to 84 years old were randomly invited. The findings of this study demonstrated that there was no difference between the two groups regarding AIx and PWV (MD = 0.0%, 95%CI: −2.4 to 2.5; MD = −0.1 m/s, 95%CI: −0.2 to 0.0, respectively) [236].

RCTs evaluating the effects of vitamin D on health outcomes differ significantly from the standard drug trials, as it is impossible to completely exclude vitamin D intake or sunlight exposure in both arms of the study. While most RCTs are conducted in the general population to enhance the generalizability of the results, it is well known that anthropometric characteristics such as age, body mass index (BMI), and skin pigmentation can influence vitamin D intake or metabolism, thereby acting as confounding factors [237]. For instance, aging can reduce the cutaneous synthesis of vitamin D. Additionally, many RCTs do not monitor baseline and in-treatment 25(OH)D levels, which further complicates the results. This is because participants in the placebo group may actually achieve higher 25(OH)D levels compared to those receiving treatment, thus introducing significant confounding [238].

The upper intake level (UL) of vitamin D set by the Food and Nutrition Board (FNB) at 50 μg (2000 IU) is considered by many to be overly conservative and not supported by current evidence [239]. Despite evidence suggesting its safety, caution has historically limited the intake to no more than 25 μg (1000 IU) per day due to potential toxicity concerns. The current UL of 50 μg (2000 IU) per day, as suggested by the Institute of Medicine (IOM), is considered too low based on substantial evidence, suggesting it may need to be increased by at least fivefold [240]. Clinical trials indicate that a daily intake of 250 μg (10,000 IU) of vitamin D3 over an extended period is unlikely to cause adverse effects in nearly all individuals in the general population, meeting the criteria for a tolerable upper intake level [241].

### 5.7. Vitamin K

A systematic review published in 2020 retrieved nine trials assessing the effectiveness of vitamin K supplementation on arterial stiffness, atherosclerosis, and calcification outcomes. Of the total nine included studies, three examined changes in PWV, and it was indicated that vitamin K supplements are not superior to the control group in ameliorating arterial stiffness markers in both the general population as well as the high-risk population, including patients with CKD or subjects aged ≥60 years old [242].

A more recent systematic review and meta-analysis of RCTs conducted by Geng et al. [243] aimed to evaluate the effect of vitamin K supplementation on arterial stiffness in patients with CKD. Ten studies were included in the review, whereas three studies were included in the meta-analysis. No association was observed between changes in PWV after vitamin K supplementation compared to the control group (MD = 0.08, 95%CI: −0.41 to 0.57).

### 5.8. Potassium

A RCT performed by Blanch et al. [244] included normotensive participants who had their FMD evaluated after the consumption of either a meal containing 36 mmol of potassium (High K) and a control meal containing 6 mmol of potassium (Low K) on two separate occasions. Regarding FMD, the investigators found a postprandial decrease following both meals. There was a notable impact of both meal (*p* < 0.05) and time (*p* < 0.01), yet there was no observed interaction between meal and time.

A meta-analysis including seven RCTs with 409 participants by Tang and colleagues [245] aimed to assess the effect of potassium supplementation compared to the control group on vascular function and concluded that there was no difference between intervention and the control group in PWV (SMD = −0.342, 95%CI: −1.123 to 0.44), AIx (SMD = −0.114, 95%CI: −0.282 to 0.054), and FMD (SMD = 0.278, 95%CI: −0.321 to 0.877).

A systematic review and meta-analysis published by D’Elia and colleagues [246] evaluated the effects of potassium supplementation on endothelial function as measured by FMD. Overall, five studies with 332 participants were included in the quantitative synthesis and it was found that potassium supplementation was associated with an improvement in FMD (MD = 0.74%, 95%CI: 0.22 to 1.25). The authors of the study also demonstrated that the observed effect was directly related to the amount of potassium supplement.

### 5.9. Multivitamin Supplementation

Harris and colleagues [247] conducted a RCT with females aged 50 years or more and males aged 50–65 years, with no underlying conditions. The supplement used in this study included vitamins, minerals, plant extracts, and probiotics that differed between genders regarding dosage and some herbal components. According to the results, no differences were observed regarding AIx%, neither for males nor for females (*p* = 0.841; *p* = 0.296, respectively).

Van Dijk and colleagues [248] assessed the effect of vitamin B12 and folic acid supplementation compared to the placebo on arterial stiffness, atherosclerosis, and cardiovascular events in an elderly population through a RCT. They randomized 2919 participants, 569 (IC: *n* = 274, PG: *n* = 295) of them comprising the vascular subgroup. AIx and PWV were the measures of arterial stiffness. After 2 years of intervention, there was no difference between the intervention and control group in both endpoints (AIx%: IG: 27.5 ± 0.6 vs. PG: 28.8 ± 0.6; PWV m/s: IG: 14.2 ± 0.3 vs. PG: 14.3 ± 0.3) [248]. 

All of the above information is summarized in Table 5.

## 6. Discussion

This literature review aimed to synthesize the published literature regarding the effects of food groups, nutraceuticals, and dietary supplements on markers of vascular health. Based on the included studies, there are mixed findings regarding the effectiveness of these substances on vascular health.

Regarding food groups, the consumption of fruits and vegetables and dairy products seems to benefit cfPWV, while nut consumption may improve FMD.

Studies examining the effect of nutraceuticals on indicators of vascular health and endothelial function yielded contradictory findings. More specifically, nitrate or nitric oxide consumption did not affect AIx, cfPWV, and FMD. As far as omega-3 fatty acid supplementation is concerned, the findings of the included studies show that it may increase FMD. Contrarily, the studies evaluating the effectiveness of CoQ10 and curcumin supplementation presented mixed findings on markers of arterial stiffness and endothelial function.

Furthermore, resveratrol or flavonoids supplementation may increase FMD and decrease PWV, improving endothelial function and arterial stiffness. As far as the effects of resveratrol on vascular health are concerned, the findings of two different systematic reviews and meta-analysis are in line, showing that resveratrol supplementation increases FMD levels [251,252]. Regarding the effects of flavonoids on vascular health, a recent systematic review and meta-analysis assessing the effects of citrus flavonoid supplementation (CFS) on endothelial function demonstrated that CFS increased FMD compared to the placebo [253]. 

The findings of a systematic review and meta-analysis showed that catechin supplementation is effective in improving AIx, FMD, and PWV. Contrarily, it did not have a beneficial effect on other markers of endothelial function, such as VCAM-1 and endothelin [165].

As far as quercetin is concerned, a systematic review and meta-analysis that assessed the effects of quercetin supplementation on endothelial function indicated that quercetin supplementation was not effective in ameliorating VCAM-1 and ICAM-1, markers of endothelial function, among patients with metabolic syndrome and related disorders [254].

The results from two RCTs, evaluating the effects of anthocyanin supplementation compared to the placebo, demonstrated that the intervention group was superior to the control group in ameliorating endothelial function markers, such as radial artery AIx, FMD, and sVCAM-1 [255,256]. The findings of the RCTs are in agreement with the findings of a systematic review and meta-analysis, which showed that anthocyanin-rich foods or extracts are effective in ameliorating FMD and PWV [170].

Finally, the majority of published studies assessing the effectiveness of various dietary supplements on vascular health indicators have shown that these supplements are not superior to placebos in improving the markers of interest.

The limitations of the included studies encompass the great clinical heterogeneity stemming from the different populations, dosages, and times of intervention in the clinical studies. As far as observational studies are concerned, flaws such as information and selection biases and confounding factors that are not properly addressed may be present, leading to findings with poor credibility [257].

More specifically, the studies that evaluated the association between food groups and vascular health outcomes involved specific populations, such as healthy individuals, hypertensive patients, or hypercholesterolemic men, which may limit the generalizability of results to broader or more diverse groups (e.g., women, racial minorities, or different socioeconomic backgrounds). Another limitation is that a significant number of studies were cross-sectional or observational, which limits the ability to draw causal relationships. Intervention durations were often short, and longer-term studies are needed to observe more significant or sustained effects. Additionally, small sample sizes in many studies increased the potential for random error and limited statistical power. Some studies had quality issues, such as inadequate randomization, lack of double-blinding, and participant dropouts, affecting the reliability of the findings. Moreover, variability in measurement methods (e.g., food frequency questionnaires, blood pressure devices) and the accuracy of dietary intake reporting can introduce errors and affect study outcomes. The misclassification of dietary measures is a common issue, leading to potential underestimation or overestimation of effects. Furthermore, small studies with significant effects are more likely to be published, introducing publication bias. Heterogeneity in study design, participant health status, and intervention types (e.g., different types of nuts, fiber sources, or fruit and vegetable interventions) complicate comparisons and generalizations. Lastly, many studies focus on specific fruits, vegetables, or components (e.g., lycopene in tomatoes, nitrates in spinach) rather than a broader dietary pattern. This focus limits the ability to draw conclusions about overall diet impacts on vascular health.

As far as the studies assessing the relationship between nutraceuticals and endothelial function and/or arterial stiffness are concerned, they involved heterogeneous populations, including participants with varying health conditions, ages, sexes, and baseline risk factors. This variability can introduce confounding factors and increase heterogeneity, making it difficult to isolate the effects of the intervention. Additionally, most studies often failed to include diverse demographics such as women, ethnic minorities, and individuals with different baseline health statuses, limiting generalizability. Moreover, cross-sectional designs were commonly used, which are limited in establishing causality. Additionally, several studies employed small sample sizes, reducing statistical power and increasing susceptibility to random error and bias. The use of short study durations also hindered the observation of long-term effects. Blinding and the use of appropriate control groups were often inconsistent, which could lead to biased outcomes. Many studies relied on self-reported dietary intake and biomarkers, which can be prone to inaccuracies and misclassification. Incomplete reporting on specific outcomes (e.g., endothelial function, plasma biomarkers) further limits the reliability of results. Variations in methodologies, such as different measurement techniques for blood pressure or biomarkers, can introduce additional sources of bias and heterogeneity. In systematic reviews and/or meta-analyses, there was evidence of publication bias, particularly in studies with small sample sizes that tend to report large effect sizes. This bias could skew the overall findings in meta-analyses, as positive results are more likely to be published than negative or neutral ones. Furthermore, differences in intervention types (e.g., nutrient doses, supplementation forms, or dietary sources) and variations in adherence to interventions (e.g., diet restrictions or supplementation protocols) could affect the study outcomes. Variability in formulation and bioavailability of supplements, such as curcumin or omega-3 fatty acids, further complicates the interpretation and comparison of results. Also, many studies did not conduct long-term follow-ups, limiting the ability to assess sustained effects or the impact of interventions on the progression of cardiovascular diseases. This lack of longitudinal data reduces the ability to make conclusive statements about the effectiveness of dietary or supplemental interventions on long-term health outcomes.

Finally, many studies that evaluated the effects of supplements on vascular health reported small sample sizes, leading to low statistical power, difficulty in performing subgroup analyses, and limitations in the generalizability of the findings. The loss of participants over time and differences in group sizes due to dropouts were also noted. The absence of proper control groups, washout periods, and blinding techniques was often cited, which could introduce biases, especially in crossover trials and studies assessing dietary or supplement interventions. A significant degree of heterogeneity in study designs, populations (age, sex, health status), and intervention protocols (doses, duration) was commonly reported. This variability, along with publication bias, complicates the generalizability and reliability of the findings. Many studies did not use the most accurate or comprehensive methods to measure key biomarkers or physiological responses, such as blood pressure, endothelial function, and arterial stiffness. For example, office blood pressure measurements were used instead of 24 h ambulatory monitoring, and some studies did not measure intracellular concentrations of relevant substances like magnesium. The short duration of many studies limited the ability to observe the long-term effects of interventions on health outcomes, such as cardiovascular events and vascular remodeling. Unmeasured factors such as dietary intake, physical activity, baseline health conditions, and interoperator variability in measurements could have influenced study outcomes. Residual confounding due to these unaccounted factors was often highlighted. The results of several studies were not generalizable due to specific participant characteristics (e.g., only men, specific ethnicities, or patients with particular health conditions), limiting the applicability of findings to broader populations, including women, children, or different ethnic groups.

## 7. Conclusions

In conclusion, this review found mixed evidence on the effectiveness of various food groups, nutraceuticals, and dietary supplements in improving vascular health, with some studies showing significant benefits and others indicating minimal or no effect. Based on the current body of the included studies, food groups, nutraceuticals, and dietary supplements may not demonstrate superiority over a placebo in enhancing markers of vascular health. To obtain more reliable evidence on the effectiveness of interventions in vascular health, additional RCTs with larger sample sizes, extended follow-up periods, and multi-center participation are necessary. Enhancing the credibility of these RCTs requires better control of dietary variables and more precise measurement of vascular health markers.

## Figures and Tables

**Table 1:** Food groups.

Reference	Study Design	Population	Interventions	Outcomes	Findings *
Hung et al., 2003 [85]	Cohort	Men initially free of CVD and diabetes, aged 40–75 years old (*n* = 44,059)	Fruit and vegetable intake	PAD diagnosis	F and V ↔ PAD
Blanch et al., 2015 [9]	SR	46 studies (four CS and 42 IS), including participants aged 18–75 years old	Fruit and vegetable intake	AIx, cfPWV, PWV, sICAM-1	↑ F and V → ↔ AS and EF
Petersen et al., 2016 [32]	RCT	Adults with T1DM and T2DM (mean age: Arm 1: 57 ± 12, Arm 2: 58 ± 12 ^†^) (*n* = 109)	Arm 1: Dietary counseling Arm 2: Usual diet	AIx (%) and cfPWV	↔ AIx (%) and cfPWV **
Bondonno et al., 2014 [37]	RCT CO	Adults with high normal BP, aged 38–70 years old (*n* = 38)	Arm 1: Green leafy vegetables (HND) Arm 2: Low-nitrate diet	AIx75 and cfPWV	↔ AIx75 and cfPWV
Jovanovski et al., 2015 [38]	RCT CO	Healthy participants (mean age 24.5 ± 11 years) (*n* = 27)	Arm1: Spinach (HND) Arm 2: Asparagus (Low-nitrate soup)	AIx75	↔ AIx75
Aatola et al., 2010 [39]	Cohort	Children aged 3–18 years old (*n* = 1622)	Fruit and vegetable intake	PWV	VC → ↓ PWV F and V → ↓ PWV
Kondo et al., 2017 [86]	RCT	P with T2DM (mean age: Arm 1: 65.2 ± 8.7, Arm 2: 68.1 ± 6.8 years ^†^) (*n* = 29)	Arm 1: Brown rice Arm 2: White rice	pFBF, FDR, RH duration	Arm 1 → ↑ EF
Brownlee et al., 2010 [87]	RCT (WHOLEheart)	P aged 18–65 years	Arm 1: 60 g WG/d for 16 weeks Arm 2: 60 g WG/d for 8 weeks and 120 g WG/d for 8 weeks Arm 3: Usual diet	e-selectin, ICAM-1, VCAM-1	↔ EF
Ribeiro et al., 2018 [50]	CS from Cohort	P in Brazil (mean age 51.7 ± 8.9 years ^†^) (*n* = 12892)	Dairy intake	cfPWV	↑ dairy intake → ↓ cfPWV compared to the lowest intake
Diez-Fernández et al., 2019 [51]	SR and MA of CS	Seven studies including adults (mean age ranged from 26.3 ± 4.2 to 63.8 ± 12.4 years ^†^) (*n* = 6) and children (13.2 ± 0.7 years ^†)^ (*n* = 1) (total n_participants_ = 16,427)	Dairy intake	PWV	Total dairy → ↓ PWV Milk → ↔ PWV Cheese → ↓ PWV
Sarapis et al., 2020 [53]	RCT CO (OLIVAUS)	Adults (age 38.5 ± 13.9 years ^†^) (*n* = 50)	Arm 1: 60 mL/d HPOO Arm 2: 60 mL/d LPOO	AΙx and PWV	↔ AIx and PWV
Sanders et al., 2011 [54]	RCT	Non-smoker adults (mean age: Arm 1: 55 (53, 56), Arm 2: 55 (54, 56), Arm 3: 55 (54, 57), Arm 4: 55 (54, 57) years ‖) (*n* = 367)	Arm 1: 0.45 g n-3 LC PUFA Arm 2: 0.9 g n-3 LC PUFA Arm 3: 1.8 g n-3 LC PUFA Arm 4: Placebo	FMD	↔ FMD between arms and placebo
Smiljanec et al., 2020 [62]	CS	Normotensive young adults (mean age 27 ± 1 years ^‡^) (*n* = 40)	UPF, MPF	AΙx and PWV	↔ AIx and PWV
Neale et al., 2017 [56]	SR and MA of RCTs	32 studies	Arm 1: Nut consumption Arm 2: Control	FMD, ICAM-1, VCAM-1	Arm 1 → ↑ FMD Arm 1 ↔ ICAM-1, VCAM-1
Xiao et al., 2018 [57]	SR and MA of RCTs	10 studies (n_participants_ = 374)	Arm 1: Nut consumption Arm 2: Control	FMD	Arm 1 → ↑ FMD
Kasliwal et al., 2015 [55]	RCT	P with mild dyslipidemia (mean age: Arm 1: 40.4 ± 8.2, Arm 2: 37.7 ± 7.6 years ^†^) (*n* = 60)	Arm 1: LSM + 80 g pistachios Arm 2: LSM	baFMD, baPWV, cfPWV	Arm 1 → ↑ baPWV and cfPWV compared to Arm 2
Kondo et al., 2014 [63]	RCT CO	Women with PM and T2DM (mean age 69.7 ± 6.6 years ^†^) (*n* = 23)	Arm 1: FBD Arm 2: Usual diet	pFBF, FDR, RH duration	↔ pFBF, FDR, RH duration
Azad et al., 2021 [66]	SR and MA	23 studies in SR and 11 in MA	Arm 1: Coffee-derived products Arm 2: Control group	FMD	Arm 1 → ↑ FMD short-term Arm 1 → ↔ FMD long-term
Vlachopoulos et al., 2005 [88]	CS	Healthy adults (mean age 41 ± 8 years old ^†^) (*n* = 228)	Coffee consumption	AΙx and PWV	Coffee consumption → ↑ AIx Coffee consumption → ↑ PWV
Uemura et al., 2013 [68]	Baseline survey of a cohort study	Japanese men, aged 35–69 years old (*n* = 540)	Coffee and Green Tea Consumption	baPWV	Coffee Consumption → ↓ baPWV Green tea consumption → ↔ baPWV
Pavão et al., 2022 [69]	RCT CO	Adults with OW and OB, aged 37.4 ± 10.0 years old ^†^ (*n* = 18)	Arm 1: Regular caffeinated coffee Arm 2: Decaffeinated coffee	AΙx75, FMD, PWV	AIx75: no difference between groups FMD: only difference when interaction of time × group was considered PWV: only difference when time was considered
Noguchi et al., 2015 [70]	RCT CO	Healthy adults (22–30 years old, mean age 23.7 ± 2.2 ^†)^ (*n* = 27)	Arm 1: Cup of caffeinated coffee Arm 2: Cup of decaffeinated coffee	RH	Arm 1 → ↑ RH compared to Arm 2
Buscemi et al., 2010 [71]	RCT CO	Healthy adults without OB (25–49 years old, mean age 31 ± 2 ^‡^) (*n* = 20)	Arm 1: Caffeinated coffee Arm 2: Decaffeinated coffee	FMD	Arm 1 → ↓ FMD compared to Arm 2
Del Giorno et al., 2022 [72]	Population-based OS	Adults, mean age 53.2 ± 13.6 years ^†^ (*n* = 1095)	Arm 1: No coffee and caffeine drinkers Arm 2: Light coffee and caffeine drinkers Arm 3: Moderate coffee and caffeine drinkers	AΙx and PWV	Arms 2 and 3 → ↓ PWV compared to Arm 1
Sasaki et al., 2013 [80]	CS	Middle-aged Japanese adults, mean age 46.5 ± 7.1 ^†^ (*n* = 812)	Women: Arm 1: Non-drinkers Arm 2: <10 g/w alcohol consumption Arm 3: 10–19 g/w alcohol consumption Arm 4: 20–29 g/w alcohol consumption Arm 5: ≥30 g/w alcohol consumption Men: Arm 1: Non-drinkers Arm 2: <20 g/w alcohol consumption Arm 3: 20–39 g/w alcohol consumption Arm 4: 40–59 g/w alcohol consumption Arm 5: ≥60 g/w alcohol consumption	baPWV	W: Arm 1 → ↑ PWV compared to Arm 2 M: Arm 1, 2, and 5 → ↑ PWV compared to Arm 3
Gonzalez-Sanchez et al., 2020 [79]	CS	Adults, mean age 55.90 ± 14.24 years ^†^ (*n* = 501)	Arm 1: Non-alcohol drinkers Arm 2: ≤30 g/w alcohol consumption Arm 3: 30–70 g/w alcohol consumption Arm 4: >70 g/w alcohol consumption	baPWV, cfPWV, cIMT	Arm 4 → ↑ cIMT and cfPWV compared to Arm 1 Arm 4 → ↔ baPWV compared to Arm 1

↑: increase; ↓: decrease; ↔: no effect. † Data are presented in mean ± standard deviation. ‡ Data are presented in mean ± standard error. ‖ Mean; 95% CI in parentheses. AIx: Augmentation Index; AS: Arterial Stiffness; baFMD: Brachial Ankle Flow-Mediated Dilatation; baPWV Brachial Ankle Pulse Wave Velocity; BP: Blood Pressure; cfPWV: Carotid–Femoral Pulse Wave Velocity; cIMT: Carotid Intima-Media Thickness; CO: Crossover; CS: Cross-Sectional; CVD: Cardiovascular Disease; EF: Endothelial Function; F: Fruits; FBD: Fish-Based Diet; FDR: Flow Debt Repayment; FMD: Flow-Mediated Dilation; HND: High Nitrate Diet; HPOO: High Polyphenol Olive Oil; ICAM-1: Intercellular Adhesion Molecule 1; LC: Long Chain; LPOO: Low Polyphenol Olive Oil; LSM: Lifestyle Modification; MA: Meta-Analysis; MPF: Minimally Processed Foods; OB: Obesity; OS: Observational Study; OW: Overweight; P: Participants; PAD: Peripheral Arterial Disease; pFBF: Peak Forearm Blood Flow; PM: Post Menopause; PUFA: Polyunsaturated Fatty Acid; PWV: Pulse Wave Velocity; RH: Reactive Hyperemia; RCT: Randomized Controlled Trial; SR: Systematic Review; T1DM: Type 1 Diabetes Mellitus; T2DM: Type 2 Diabetes Mellitus; TD: Treatment Duration; UPF: Ultra-Processed Food; V: Vegetables; VC: Vegetable Consumption; VCAM-1: Vascular Cell Adhesion Molecule 1; WG: Whole Grain. * In the RCTs, the findings are reported compared to the control group, unless otherwise stated. ** time-by treatment effect.

**Table 2:** Study characteristics including nitrate or nitric oxide as an intervention group.

Reference	Study Design	Population	Interventions	Outcomes	Findings *
Bahrami et al., 2021 [100]	SR and MA of RCTs	27 RCTs (n_participants_ = 765)	Arm 1: nitrate supplementation Arm 2: placebo/control	AIx, FMD and PWV	↔ AIx ↑ FMD ↓ PWV
Webb et al., 2008 [90]	RCT CO	Healthy adults (mean age 26.6 ± 7.4 years ^‡^) (*n* = 10)	Arm 1: 500 mL beetroot juice 2 h before I/R Arm 2: no treatment	FMD	↑ FMD
Kapil et al., 2010 [99]	RCT CO	Healthy adults (mean age 24.7 ± 1.4 years ^‡^) (*n* = 12)	Arm 1: KNO_3_ Arm 2: KCl Arm 3: Beetroot juice	FMD	↓ FMD response
Joris et al., 2013 [95]	RCT CO	Healthy men, 61 ± 7 years ^†^ (*n* = 20)	Arm 1: Two muffins (56.6 g fat) and 140 mL of concentrated beetroot juice (500 mg dietary nitrate) Arm 2: Two muffins (56.6 g fat) and 140 mL control drink	baFMD, CAIx, CAIxHR75, cfPWV, PAIx	↓ FMD ↔ CAIx, CAIxHR75, PAIx, cfPWV
Zafeiridis et al., 2019 [97]	RCT CO	Drug-naive patients with hypertension, aged 44.0 ± 2.6 years ^‡^	Arm 1: Nitrate-rich beetroot juice Arm 2: Nitrate-depleted beetroot juice	cfPWV	↔ cfPWV
Floyd et al., 2019 [94]	RCT CO	Healthy adults, aged 27.1 ± 6.5 years ^†^ (*n* = 33)	Arm 1: KNO_3_ Arm 2: placebo (KCl) Two independent cohorts: Cohort 1: high-dose (24 mmol) Cohort 2: low-dose (8 mmol) 1 h prior to an oral glucose tolerance test	AIx and PWV	↔ AIx, BP, HR, PWV Subgroup analysis: ↔ ΔPWV, ΔcSBP or ΔAIx for the interventions within either cohort
Mayra et al., 2019 [96]	Pilot RCT	Postmenopausal women, aged 52.6 ± 4.8 years ^†^ (*n* = 10)	Arm 1: two high-nitrate salads/day for 10 days (284 mg/d nitrate) Arm 2: low-nitrate, canned vegetable control (beans, corn, or peas)	FMD	↑ FMD
Walker et al., 2019 [93]	RCT CO	Healthy males (mean 69 ± 4 years ^†^) (*n* = 15)	Arm 1: 140 mL NO_3_^−^-rich beetroot juice Arm 2: 150 mL placebo drink	AIx, FMD, PWV	↔ PWV ↓ AIx ↑ FMD
Morselli et al., 2021 [98]	RCT	P with or at risk of T2DM (mean age: Arm 1: 56.6 ± 15, Arm 2: 57.8 ± 14.6, Arm 3: 57.4 ± 12.1, Arm 4: 59.1 ± 11.3 ^†^) (*n* = 93)	Arm 1: nitrate-containing beetroot juice and spironolactone Arm 2: nitrate-containing beetroot juice and doxazosin Arm 3: placebo nitrate-depleted juice and spironolactone Arm 4: placebo nitrate-depleted juice and doxazosin	cIMT	↓ cIMT (Nitrate-containing juice vs. placebo juice)

↑: increase; ↓: decrease; ↔: no effect. * In the RCTs, the findings are reported compared to the control group, unless otherwise stated. † Data are presented in mean ± standard deviation. ‡ Data are presented in mean ± standard error. AIx: Augmentation Index; baFMD: Brachial Ankle Flow-Mediated Dilatation; baPWV Brachial Ankle Pulse Wave Velocity; cAIx: Central Augmentation Index; CAIxHR75: central AIx adjusted for heart rate; cfPWV: Carotid–Femoral Pulse Wave Velocity; cIMT: Carotid Intima-Media Thickness; CO: Crossover; FMD: Flow-Mediated Dilation; MA: Meta-Analysis; P: Participants; PAIx: Peripheral Augmentation Index; PWV: Pulse Wave Velocity; RCT: Randomized Controlled Trial; SBP: Systolic Blood Pressure; SR: Systematic Review; T2DM: Type 2 Diabetes Mellitus.

**Table 3:** Study characteristics including resveratrol, flavonoids, quercetin, and tea catechins as a dietary intervention.

Reference	Study Design	Population	Interventions	Outcomes	Findings *
Hooper et al., 2008 [126]	SR and MA of RCTs	133 RCTs and 6557 participants	Different types of flavonoids	FMD	↑ FMD (chocolate/cocoa, soy protein isolate, isoflavone extract, black tea) ↓ FMD (flavonols).
Fairlie-Jones et al., 2017 [170]	SR and MA of RCTs	26 studies in the SR 24 studies in the MA Mean age range 20.8–70.1 years	Anthocyanin-rich foods or extracts Arm 1: Acute Supplementation Arm 2: Chronic Supplementation	FMD and PWV	Arm 1 and 2 → ↑ FMD, Arm 1 → ↓ PWV
Shafabakhsh et al., 2020 [165]	SR and MA of RCTS	16 studies with 22 effect sizes	Arm 1: Catechin supplementation Arm 2: Placebo/control	FMD and PWV	↑ FMD ↓ AIx, PWV
Jochmann et al., 2007 [160]	RCT CO	Healthy postmenopausal women (mean age 58.7 ± 4.5 years ^†^) (*n* = 21)	Arm 1: 500 mL boiled water Arm 2: 500 mL black tea Arm 3: 500 mL green tea	FMD and NMD	↔ FMD between green and black tea (but ingestion of green and black tea led to ↑ FMD) ↔ NMD between all groups
Grassi et al., 2009 [161]	RCT CO	Healthy males (mean age 32.9 ± 10.2 years ^†^) (*n* = 19)	Arm 1: 0 mg tea flavonoids Arm 2: 100 mg tea flavonoids Arm 3: 200 mg tea flavonoids Arm 4: 400 mg tea flavonoids Arm 5: 800 mg tea flavonoids	FMD and PWV	↑ FMD (dose-dependent relationship) ↔ PWV
Wong et al., 2010 [119]	RCT	Men and postmenopausal women with untreated borderline hypertension (mean age 55 ± 2 years ^‡^) (*n* = 19)	Arm 1: 30 mg RSV Arm 2: 90 mg RSV Arm 3: 270 mg RSV Arm 4: Placebo	FMD	↑ FMD (dose-response relationship)
Barona et al., 2012 [132]	RCT	Men with metabolic syndrome (mean age 51.3 ± 9.6 years ^†^) (*n* = 24)	Arm 1: Freeze-dried grape polyphenol powder (GRAPE) Arm 2: Placebo	FMD	↑ FMD
Siasos et al., 2013 [135]	RCT CO	Healthy smokers (mean age 26.34 ± 4.93 years ^†^) (*n* = 26)	Arm 1: Concord grape juice Arm 2: Placebo (grapefruit juice)	cfPWV and FMD	↓ cfPWV ↑ FMD
Wong et al., 2013 [116]	RCT	Healthy adults with obesity (mean age 61 ± 1.3 years ^‡^) (*n* = 28)	Arm 1: 75 mg capsule of trans-resveratrol Arm 2: Placebo	FMD	↑ FMD
Choi et al., 2015 [144]	RCT	Individuals with overweight and obesity (mean age: Arm 1: 43.6 ± 9.1, Arm 2: 42.5 ± 8.9 years ^†^) (*n* = 72)	Arm 1: Onion peel extract (100 mg quercetin/d) Arm 2: Placebo (identical capsule)	EPCs and FMD	↑ EPCs ↑ FMD
Draijer et al., 2015 [134]	RCT	Subjects with mild hypertension (mean age 57.6 ± 9.9 years) (*n* = 60)	Arm 1: Capsules with grape juice extract (high concentrations of anthocyanins and flavonols, but poor in catechins and procyanidins) Arm 2: Capsules with a mixture of grape and wine extract (high concentrations of anthocyanins and flavonols) Arm 3: Placebo capsules	FMD	↔ FMD
Heiss et al., 2015 [127]	RCT	Young (<35 years, *n* = 20) and elderly (50–80 years, *n* = 20) healthy male Caucasian adult subjects	Arm 1: Cocoa flavanol-containing drink Arm 2: Nutrient-matched cocoa flavanol-free drink	AIx, baFMD, FMD, NMD, PWV	↑ baFMD (Arm 1 compared to baseline in both young and elderly) ↓ PWV in elderly and young ↓ AIx only in elderly ↔ NMD
Sansone et al., 2015 [128]	Pilot RCT	Healthy, middle-aged (35–60 years) men and women (*n* = 100)	Arm 1: CF-containing drink (450 mg) Arm 2: Nutrient-matched CF-free drink	FMD and PWV	↑ FMD ↓ PWV
Vaisman et al., 2015 [133]	RCT	Subjects with prehypertension and mild hypertension (mean age: Arm 1: 58.5 ± 7.9, Arm 2: 57.6 ± 7.2, Arm 3: 56.4 ± 7.0 years ^†^) (*n* = 50)	Arm 1: 200 mg RGC Arm 2: 400 mg RGC Arm 3: Placebo	FMD	↑ FMD
Tsang et al., 2018 [169]	RCT CO	Healthy adults, aged 20–55 years (*n* = 14)	Arm 1: 200 g/day of cooked purple potato containing 288 mg anthocyanins Arm 2: 200 g/day white potato containing negligible anthocyanins	PWV	Arm 2 → ↓ PWV compared to baseline Arm 1 →↔ PWV compared to baseline
Diaz et al., 2020 [118]	Placebo-controlled, single-blind, crossover design	P with CAD aged 66.6 ± 7.8 years ^†^ (*n* = 10)	Arm 1: RSV Supplementation Arm 2: Placebo	FMD	↑ FMD in patients after CABG surgery ↓ FMD in patients who underwent PCI
Odai et al., 2019 [131]	RCT	P with prehypertension, aged 53.7 ± 7.7 years old ^†^ (*n* = 30)	Arm 1: Low-dose (200 mg/day) GSPE Arm 2: High-dose (400 mg/day) GSPE Arm 3: Placebo	FMD and PWV	↔ FMD ↓ PWV in the high-dose group
Garcia-Yu et al., 2020 [130]	RCT	Postmenopausal women (mean age: Arm 1: 57.1 ± 3.5, Arm 2: 57.5 ± 3.8 years ^†^) (*n* = 140)	Arm 1: Chocolate (99% cocoa) Arm 2: Control (no intervention)	baPWV	↔ baPWV
Gröne et al., 2020 [129]	RCT	Young (<35 years, *n* = 20) and elderly (50–80 years, *n* = 19) healthy male Caucasian adult subjects	Arm 1: Cocoa flavanol-containing drink (450 mg) Arm 2: Nutrient-matched cocoa flavanol-free drink	EMPs	↓ EMPs
Gonçalinho et al., 2021 [117]	RCT	Healthy adults (mean age: Arm 1: 58.5 ± 3.4, Arm 2: 58.6 ± 3.6 ^†^) (*n* = 48)	Arm 1: RSV supplementation Arm 2: ER	FMD	↔ FMD
Arisi et al., 2023 [171]	RCT	Volunteers with excess weight (mean age 38.8 ± 6.4 years old ^†^) (*n* = 55)	Arm 1: Individualized meal plan and açaí–juçara 200 g Arm 2: Individualized meal plan	FMW and PWV	↔ FMD ↓ PWV

↑: increase; ↓: decrease; ↔: no effect. * In the RCTs, the findings are reported compared to the control group, unless otherwise stated. † Data are presented in mean ± standard deviation. ‡ Data are presented in mean ± standard error. AIx: Augmentation Index; baFMD: Brachial Ankle Flow-Mediated Dilatation; baPWV Brachial Ankle Pulse Wave Velocity; BP: Blood Pressure; CABG: Coronary Artery Bypass Grafting; CAD: Coronary Artery Disease; CF: Cocoa Flavanols; CO: Crossover; EMPs: Endothelial Microparticles; EPCs: Endothelial Progenitor Cells; ER: Energy Restriction; FBF: Forearm Blood Flow; FMD: Flow-Mediated Dilation; GSPE: Grape Seed Proanthocyanidin Extract; NMD: Nitro-Mediated Dilatation; P: Participants; PCI: Percutaneous Coronary Intervention; PWV: Pulse Wave Velocity; RCT: Randomized Controlled Trial; RGC: Red Grape Cell; RSV: Resveratrol.

**Table 4:** Study characteristics including coenzyme Q10, antioxidants, curcumin, omega-3 fatty acids, and selenium as a dietary intervention.

Reference	Study Design	Population	Interventions	Outcomes	Findings *
Gao et al., 2012 [177]	SR and MA of RCTs	Patients with and without established cardiovascular disease, mean age ranged from 34.0 to 68.9 years (5 RCTs—194 patients)	Arm 1: CoQ10 Arm 2: Placebo	FMD	↑ FMD
Wang et al., 2012 [198]	SR and MA of RCTs	16 eligible studies involving 901 participants, mean age range 9.0 to 66.0 years	Arm 1: Omega-3 FAs Arm 2: Placebo	FMD	↑ FMD
Lee et al., 2023 [192]	SR and MA of RCTs	38 studies in the MA	Arm 1: Omega-3 FAs Arm 2: Control	cIMT and FMD	↑ FMD ↔ c-IMT
Hamilton et al., 2009 [176]	RCT	Patients with T2DM, aged 68 ± 6 years ^†^ (*n* = 23)	Arm 1: Oral CoQ10 (200 mg/day) Arm 2: Placebo	FMD and NMD	↑ FMD ↔ NMD
Żebrowska et al., 2015 [197]	RCT	Endurance-trained athletes, aged 23.1 ± 5.4 years ^†^ (*n* = 13)	Arm 1: Omega-3 FAs supplementation Arm 2: Placebo	FMD, NMD, PWV	↑ FMD ↔ NMD ↔ PWV
Sawada et al., 2016 [193]	RCT	Newly diagnosed impaired glucose metabolism patients with coronary artery disease (mean age: Arm 1: 67.8 ± 9.1, Arm 2: 68.9 ± 8.8 years old ^†^) (*n* = 107)	Arm 1: 1800 mg/day of EPA Arm 2: no EPA	FMD	↑ FMD
Casanova et al., 2017 [196]	RCT CO	Hypertensive patients with hypertriglyceridemia and high cardiovascular risk, mean age 54 years (*n* = 29)	Arm 1: Omega-3 (1800 mg/day) Arm 2: Ciprofibrate (100 mg/day)	AIx, FMD, cfPWV	Arm 1 → ↑ FMD in high-risk patients from baseline (11.1 ± 1.6 vs. 13.5 ± 1.2, *p* = 0.01) Arm 2 → ↓ cfPWV in high-risk patients from baseline (10.4 ± 0.4 vs. 9.4 ± 0.3, *p* = 0.021) ↔ AIx for both arms
Santos-Parker et al., 2017 [185]	RCT	Healthy men and postmenopausal women (mean age: Arm 1: 63 ± 2, Arm 2: 61 ± 2 years old ^‡^) (*n* = 39)	Arm 1: Curcumin (2000 mg/day) Arm 2: Placebo	FMW and PWV	↑ FMD ↔ PWV
Kawashima et al., 2020 [175]	RCT CO	Patients with heart failure with reduced ejection fraction (mean age 70 ± 9 years old ^†^) (*n* = 14)	Arm 1: CoQ10 (400 mg/day) Arm 2: Placebo	RHI	↑ RHI
O’Mahoney et al., 2020 [200]	RCT	People with T1DM (mean age: Arm 1: 32 ± 12, Arm 2: 36 ± 17 years old ^†^) (*n* = 27)	Arm 1: 3.3 g/day of encapsulated n-3 PUFA Arm 2: Placebo	cIMT and FMD	↔ cIMT and FMD
Meital et al., 2020 [194]	RCT	Men with abdominal aortic aneurysm (mean age: Arm 1: 73.6 ± 5.0, Arm 2: 75.1 ± 5.7 years old ^†^) (*n* = 30)	Arm 1: LC n-3 PUFA capsules Arm 2: Placebo	AIx and PWV	↓ PWV ↔ AIx
Alidadi et al., 2021 [186]	RCT	Patients with metabolic syndrome (mean age: Arm 1: 42.84 ± 6.25, Arm 2: 44.43 ± 5.92 ^†^) (*n* = 66)	Arm 1: Curcumin (500 mg/day) Arm 2: Placebo	AIx, AIx75, cfPWV	↓ cfPWV ↔ AIx and AIx75
Petersen et al., 2010 [199]	Observational study	Healthy individuals (*n* = 40)	Dietary intake of n-3 polyunsaturated acids	FMD	Fish intake and EPA, DHA, supplementation → ↔ FMD
Swart et al., 2019 [209]	Prospective cohort study (South African leg of the PURE study)	Black adults from rural and urban areas in South Africa (*n* = 690)	Serum selenium levels	cIMT & cfPWV	Positive association between cIMT and selenium (β = 0.12, 95%CI: 0.05 to 0.19, *p* = 0.001) No association between cfPWV and selenium (β = −0.05, 95%CI: −0.12 to 0.02, *p* = 0.163)
Gao et al., 2021 [174]	Observational study	Patients undergoing hemodialysis, median age 65 (56–73) ^◊^ (*n* = 111)	CoQ10 levels	FMD	Correlation between FMD and plasma CoQ10 level (r = 0.727, *p* < 0.001)
Maruyama et al., 2022 [195]	Prospective cohort study (the Toon Health Study)	Men and women aged 30–84 years (*n* = 1843)	Fish and omega-3 FA intake	cIMT	1. Fish intake → no association with severely increased cIMT (OR = 0.59, 95%CI: 0.33 to 1.05) 2. FA intake → lower odds of severely increased cIMT (OR = 0.55, 95%CI: 0.31 to 0.98)

↑: increase; ↓: decrease; ↔: no effect. * In the RCTs, the findings are reported compared to the control group, unless otherwise stated. † Data are presented in mean ± standard deviation. ‡ Data are presented in mean ± standard error. ◊ Data are presented in the median (interquartile range). AIx: Augmentation Index; baFMD: Brachial Ankle Flow-Mediated Dilatation; baPWV Brachial Ankle Pulse Wave Velocity; BP: Blood Pressure; CF: Cocoa Flavanols; cfPWV: Carotid–Femoral Pulse Wave Velocity; CO: Crossover; CS: Cross-Sectional; cIMT: Carotid Intima-media Thickness; EMPs: Endothelial Microparticles; EPCs: Endothelial Progenitor Cells; EVR: Elastic Vascular Resistance; FAs: Fatty Acids; FBF: Forearm Blood Flow; FMD: Flow-Mediated Dilation; MA: Meta-Analysis; NMD: Nitro-Mediated Dilatation; PWV: Pulse Wave Velocity; RCT: Randomized Controlled Trial; RHI: Reactive Hyperemia Index; SR: Systematic Review; T1DM: Type 1 Diabetes Mellitus; T2DM: Type 2 Diabetes Mellitus.

**Table 5:** Study characteristics including dietary supplementation as intervention group.

Reference	Study Design	Population	Interventions	Outcomes	Findings *
Marques et al., 2020 [215]	SR and MA of RCTs	Five RCTs (mean age range 26.3–68 years old)	Arm 1: Mg supplementation Arm 2: Placebo	PWV, FMD	↔ FMD subgroup analysis: ↑ FMD in studies longer than 6 months, in unhealthy population over 50 years old, and in those with higher BMI
de Bree et al., 2007 [224]	SR and MA of RCTs	14 RCTs (mean age range 29.3 to 69.1 years) (*n* = 732)	Arm 1: Folic Acid Supplementation Arm 2: Vitamin B6 Supplementation Arm 3: Vitamin B12 Supplementation Arm 4: Placebo	FMD	↑ FMD (folic acid vs. placebo)
D’Elia et al., 2023 [246]	SR and MA of RCTs	5 RCTS (age range 18–75 years old) (*n* = 332)	Arm 1: K supplementation Arm 2: Placebo	FMD	↑ FMD
Rodríguez et al., 2016 [232]	SR and MA of RCTs	18 studies in SR and 13 in MA (mean age range: Arm 1: 16.5–79.3, Arm 2: 16.3–80.5)	Arm 1: Vitamin D supplementation Arm 2: Placebo	PWV, AI	↔ PWV, AI
Tang et al., 2017 [245]	MA of RCTs	Seven studies (mean age range 26 to 65.8 years) (*n* = 409)	Arm 1: K supplementation Arm 2: Placebo	FMD, PWV, AI	↔ FMD, PWV, AI
Geng et al., 2023 [243]	SR and MA of RCTs	10 RCTs (*n* = 733)	Arm 1: Vitamin K supplementation Arm 2: Placebo	PWV	↔ PWV
Vlasschaert et al., 2020 [242]	SR of RCTs	Nine studies (mean age range 55–80 years) (*n* = 1589)	Arm 1: Vitamin K1 or vitamin K2 supplementation Arm 2: Placebo	PWV	↔ PWV
Zamani et al., 2023 [225]	SR and MA of RCTs	21 RCTs (mean age range 26–66 years) (*n* = 2025)	Arm 1: Folic Acid Supplementation Arm 2: Placebo	FMD	↑ FMD
Shechter et al., 2000 [212]	RCT	P with stable CAD (age range 42 to 82 years) (*n* = 50)	Arm 1: Magnesium supplementation Arm 2: Placebo	FMD	↑ FMD
Mullan et al., 2002 [231]	RCT	P with T2DM (mean age: Arm 1: 61.0 ± 6.5, Arm 2: 57.9 ± 6.6 years old ^†^) (*n* = 30)	Arm 1: Ascorbic acid (500 mg) Arm 2: Placebo	AIx%	↓ AIx%
Witham et al., 2012 [234]	RCT	P who had undergone stroke (mean age: Arm 1: 66.2 ± 13.0, Arm 2: 67.7 ± 6.9 years old ^†^)	Arm 1: Vitamin D (100,000 units) Arm 2: Placebo	FMD	Arm 1 → ↑ FMD at 8 weeks
Blanch et al., 2014 [244]	RCT CO	Normotensive participants (mean age 31 ± 11 years old ^†^) (*n* = 32)	Arm 1: 36 mmol potassium (High K) Arm 2: 6 mmol potassium (Low K)	FMD	Arm 1 → ↓ FMD Arm 2 → ↓ FMD Arm 1 ↓ FMD less than Arm 2 (*p* < 0.05)
Witham et al., 2013 [233]	RCT	Healthy women (mean age: Arm 1: 41.7 ± 13.4, Arm 2: 39.4 ± 11.8 years old ^†^) (*n* = 50)	Arm 1: Vitamin D supplementation Arm 2: Placebo	FMD	↔ FMD
Knapen et al., 2015 [249]	RCT	Healthy women (mean age 59.5 ± 3.3 years old ^†^) (*n* = 244)	Arm 1: MK-7 Arm 2: Placebo	cfPWV and crPWV	MK-7 → ↑ Arterial Stiffness
Van Dijk et al., 2015 [248]	RCT	Older adults (mean age: Arm 1: 74 ± 6.6, Arm 2: 74.2 ± 6.4 years old ^†^) (*n* = 2919)	Arm 1: Vitamin B12 and folic acid Arm 2: Placebo	AIx, cIMT, and PWV	↔ AIx, cIMT, PWV
Joris et al., 2016 [213]	RCT	Adults with overweight or obesity (mean age: Arm 1: 62 ± 5, Arm 2: 62 ± 6 years old ^†^) (*n* = 52)	Arm 1: Mg supplementation Arm 2: Placebo	CAIxHR75% and cfPWV	↓ cfPWV ↔ CAIxHR75%
Harris et al., 2016 [247]	RCT	Healthy older people (mean age: Arm 1: female: 64.2 ± 7.4, male: 62.0 ± 6.5, Arm 2: female: 62.6 ± 7.0, male: 60.6 ± 5.6 years old ^†^) (*n* = 239)	Arm 1: Multivitamin supplementation Arm 2: Placebo	AIx%	↔ AIx%
Kumar et al., 2017 [235]	RCT	P with CKD (mean age: Arm 1: 43.17 ± 11.79, Arm 2: 45.20 ± 11.61 years ^†^) (*n* = 120)	Arm 1: Cholecalciferol supplementation Arm 2: Placebo	FMD and cfPWV	↑FMD ↓ cfPWV
Sluyter et al., 2017 [236]	RCT	Adults (mean age: Arm 1: 64.6 ± 8.4, Arm 2: 65.4 ± 9.0 years ^†^) (*n* = 517)	Arm 1: Vitamin D supplementation Arm 2: Placebo	AIx and PWV	↔ AIx 7 PWV
Maruyama et al., 2019 [222]	RCT CO	Japanese adults with at least one component of metabolic syndrome without medication (mean age: early intervention group: 53.9 ± 6.1, late intervention group: 53.2 ± 6.8 years ^†^) (*n* = 127)	Arm 1: Supplement drink with vitamins B6, B12, and C and folate (early intervention group) Arm 2: Supplement drink with vitamin B6, B12, C and folate (late intervention group)	FMD	↔ FMD
Dalan et al., 2020 [250]	RCT	P with haptoglobin genotype diabetes (mean age 56 ± 10 years ^†^) (*n* = 187)	Arm 1: Vitamin E Arm 2: Placebo	cIMT and PWV	Vitamin E ↔ cIMT and PWV
Schutten et al., 2022 [214]	RCT	Overweight and slightly obese subjects (mean age 63.2 ± 6.8 years ^†^) (*n* = 164)	Arm 1: Magnesium citrate Arm 2: Magnesium oxide Arm 3: Magnesium sulfate Arm 4: Placebo	AIxHR75% and cfPWV	↔ cfPWV and AI@HR75%

↑: increase; ↓: decrease; ↔: no effect. * In the RCTs, the findings are reported compared to the control group, unless otherwise stated. † Data are presented in mean ± standard deviation. AASIx: Ambulatory Arterial Stiffness Index; AIx: Augmentation Index; AO: Abdominal Obesity; baPWV: Brachial Ankle Pulse Wave Velocity; cAIx75: Central Augmentation Index75; CAIxHR75%: Central Augmentation Index adjusted for heart rate; CDK: Chronic Kidney Disease; cIMT: Carotid Intima-Media Thickness; cfPWV: Carotid–Femoral Pulse Wave Velocity; CHD: Coronary Heart Disease; CHF: Chronic Heart Failure; CO: Crossover; CS: Cross-Sectional; CVD: Cardiovascular Disease; EF: Endothelial Function; EVA: Early vascular Aging; FMD: Flow-Mediated Dilation; K: Potassium; MA: Meta-Analysis; Mg: Magnesium; PCA: Principal Component Analysis; PWV: Pulse Wave Velocity; cfPWV: Carotid–Femoral Pulse Wave Velocity; rAIx75: Radial Augmentation Index75; RCT: Randomized Controlled Trial; SNP: Sodium Nitroprusside; SR: Systematic Review; T2DM: Type 2 Diabetes Mellitus; VAI: Vascular Arterial Index.

## Data Availability

Data are contained within the article.

## References

[1] World Health Organization Aging and Health. https://www.who.int/news-room/fact-sheets/detail/ageing-and-health.

[2] Kozakova M., Palombo C. (2021). Vascular Ageing and Aerobic Exercise. Int. J. Environ. Res. Public Health.

[3] Poredos P., Poredos A.V., Gregoric I. (2021). Endothelial Dysfunction and Its Clinical Implications. Angiology.

[4] González L.D.M., Romero-Orjuela S.P., Rabeya F.J., del Castillo V., Echeverri D. (2023). Age and Vascular Aging: An Unexplored Frontier. Front. Cardiovasc. Med..

[5] Niiranen T.J., Lyass A., Larson M.G., Hamburg N.M., Benjamin E.J., Mitchell G.F., Vasan R.S. (2017). Prevalence, Correlates, and Prognosis of Healthy Vascular Aging in a Western Community-Dwelling Cohort. Hypertension.

[6] Al-Qaisi M. (2008). Measurement of Endothelial Function and Its Clinical Utility for Cardiovascular Risk. Vasc. Health Risk Manag..

[7] Gomez-Sanchez M., Gomez-Sanchez L., Patino-Alonso M.C., Cunha P.G., Recio-Rodriguez J.I., Alonso-Dominguez R., Sanchez-Aguadero N., Rodriguez-Sanchez E., Maderuelo-Fernandez J.A., Garcia-Ortiz L. (2020). Vascular Aging and Its Relationship with Lifestyles and Other Risk Factors in the General Spanish Population: Early Vascular Ageing Study. J. Hypertens..

[8] Kucharska-Newton A.M., Stoner L., Meyer M.L. (2019). Determinants of Vascular Age: An Epidemiological Perspective. Clin. Chem..

[9] Blanch N., Clifton P.M., Keogh J.B. (2015). A Systematic Review of Vascular and Endothelial Function: Effects of Fruit, Vegetable and Potassium Intake. Nutr. Metab. Cardiovasc. Dis..

[10] El Assar M., Angulo J., Rodríguez-Mañas L. (2013). Oxidative Stress and Vascular Inflammation in Aging. Free Radic. Biol. Med..

[11] Sharifi-Rad M., Anil Kumar N.V., Zucca P., Varoni E.M., Dini L., Panzarini E., Rajkovic J., Tsouh Fokou P.V., Azzini E., Peluso I. (2020). Lifestyle, Oxidative Stress, and Antioxidants: Back and Forth in the Pathophysiology of Chronic Diseases. Front. Physiol..

[12] Kantor E.D., Lampe J.W., Kratz M., White E. (2013). Lifestyle Factors and Inflammation: Associations by Body Mass Index. PLoS ONE.

[13] Kakaletsis N., Kotsis V., Protogerou A.D., Vemmos K., Korompoki E., Kollias A., Karagiannis T., Milionis H., Ntaios G., Savopoulos C. (2024). Early Vascular Aging in Acute Ischemic Stroke: A Systematic Review and Meta-Analysis. J. Stroke Cerebrovasc. Dis..

[14] Filippou C.D., Tsioufis C.P., Thomopoulos C.G., Mihas C.C., Dimitriadis K.S., Sotiropoulou L.I., Chrysochoou C.A., Nihoyannopoulos P.I., Tousoulis D.M. (2020). Dietary Approaches to Stop Hypertension (DASH) Diet and Blood Pressure Reduction in Adults with and without Hypertension: A Systematic Review and Meta-Analysis of Randomized Controlled Trials. Adv. Nutr..

[15] Szczerba E., Barbaresko J., Schiemann T., Stahl-Pehe A., Schwingshackl L., Schlesinger S. (2023). Diet in the Management of Type 2 Diabetes: Umbrella Review of Systematic Reviews with Meta-Analyses of Randomised Controlled Trials. BMJ Med..

[16] Rossman M.J., LaRocca T.J., Martens C.R., Seals D.R. (2018). Healthy Lifestyle-Based Approaches for Successful Vascular Aging. J. Appl. Physiol..

[17] LaRocca T.J., Martens C.R., Seals D.R. (2017). Nutrition and Other Lifestyle Influences on Arterial Aging. Ageing Res. Rev..

[18] Man A.W.C., Li H., Xia N. (2020). Impact of Lifestyles (Diet and Exercise) on Vascular Health: Oxidative Stress and Endothelial Function. Oxid. Med. Cell. Longev..

[19] Tucci M., Marino M., Martini D., Porrini M., Riso P., Del Bo’ C. (2022). Plant-Based Foods and Vascular Function: A Systematic Review of Dietary Intervention Trials in Older Subjects and Hypothesized Mechanisms of Action. Nutrients.

[20] Theodoridis X., Chourdakis M., Papaemmanouil A., Chaloulakou S., Georgakou A.V., Chatzis G., Triantafyllou A. (2024). The Effect of Diet on Vascular Aging: A Narrative Review of the Available Literature. Life.

[21] Katsiki N., Filippatos T., Vlachopoulos C., Panagiotakos D., Milionis H., Tselepis A., Garoufi A., Rallidis L., Richter D., Nomikos T. (2023). Hellenic Atherosclerosis Society Guidelines for the Diagnosis and Treatment of Dyslipidemias—2023.

[22] Cobos-Palacios L., Ruiz-Moreno M.I., Muñoz-Ubeda M., López-Sampalo A., Vilches-Perez A., Vargas-Candela A., Benitez-Porres J., Navarro-Sanz A., Pérez-Belmonte L.M., Lopez-Carmona M.D. (2022). A Healthy Lifestyle Is Associated with Lower Arterial Stiffness in a Metabolically Healthy Elderly Population with Overweight or Obesity. J. Hypertens..

[23] Tyrovola D., Soulaidopoulos S., Tsioufis C., Lazaros G. (2023). The Role of Nutrition in Cardiovascular Disease: Current Concepts and Trends. Nutrients.

[24] American Heart Association The American Heart Association Diet and Lifestyle Recommendations. https://www.heart.org/en/healthy-living/healthy-eating/eat-smart/nutrition-basics/aha-diet-and-lifestyle-recommendations.

[25] Wang S., Melnyk J.P., Tsao R., Marcone M.F. (2011). How Natural Dietary Antioxidants in Fruits, Vegetables and Legumes Promote Vascular Health. Food Res. Int..

[26] Stanek A., Grygiel-Górniak B., Brożyna-Tkaczyk K., Myśliński W., Cholewka A., Zolghadri S. (2023). The Influence of Dietary Interventions on Arterial Stiffness in Overweight and Obese Subjects. Nutrients.

[27] Monsalve B., Concha-Meyer A., Palomo I., Fuentes E. (2017). Mechanisms of Endothelial Protection by Natural Bioactive Compounds from Fruit and Vegetables. An. Acad. Bras. Cienc..

[28] Zhan J., Liu Y.-J., Cai L.-B., Xu F.-R., Xie T., He Q.-Q. (2017). Fruit and Vegetable Consumption and Risk of Cardiovascular Disease: A Meta-Analysis of Prospective Cohort Studies. Crit. Rev. Food Sci. Nutr..

[29] Aune D., Giovannucci E., Boffetta P., Fadnes L.T., Keum N., Norat T., Greenwood D.C., Riboli E., Vatten L.J., Tonstad S. (2017). Fruit and Vegetable Intake and the Risk of Cardiovascular Disease, Total Cancer and All-Cause Mortality—A Systematic Review and Dose-Response Meta-Analysis of Prospective Studies. Int. J. Epidemiol..

[30] Blekkenhorst L., Sim M., Bondonno C., Bondonno N., Ward N., Prince R., Devine A., Lewis J., Hodgson J. (2018). Cardiovascular Health Benefits of Specific Vegetable Types: A Narrative Review. Nutrients.

[31] Theodoridis X., Chourdakis M., Chrysoula L., Chroni V., Tirodimos I., Dipla K., Gkaliagkousi E., Triantafyllou A. (2023). Adherence to the DASH Diet and Risk of Hypertension: A Systematic Review and Meta-Analysis. Nutrients.

[32] Petersen K., Clifton P., Lister N., Keogh J. (2016). Effect of Improving Dietary Quality on Arterial Stiffness in Subjects with Type 1 and Type 2 Diabetes: A 12 Months Randomised Controlled Trial. Nutrients.

[33] Zhou D.-D., Luo M., Shang A., Mao Q.-Q., Li B.-Y., Gan R.-Y., Li H.-B. (2021). Antioxidant Food Components for the Prevention and Treatment of Cardiovascular Diseases: Effects, Mechanisms, and Clinical Studies. Oxid. Med. Cell. Longev..

[34] Armoza A., Haim Y., Basiri A., Wolak T., Paran E. (2013). Tomato Extract and the Carotenoids Lycopene and Lutein Improve Endothelial Function and Attenuate Inflammatory NF-ΚB Signaling in Endothelial Cells. J. Hypertens..

[35] Robert L., Narcy A., Rock E., Demigne C., Mazur A., Rémésy C. (2006). Entire Potato Consumption Improves Lipid Metabolism and Antioxidant Status in Cholesterol-Fed Rat. Eur. J. Nutr..

[36] Eman A.M., Ferns G.A. (2017). Dietary Fruits and Vegetables and Cardiovascular Diseases Risk. Crit. Rev. Food Sci. Nutr..

[37] Bondonno C.P., Liu A.H., Croft K.D., Ward N.C., Yang X., Considine M.J., Puddey I.B., Woodman R.J., Hodgson J.M. (2014). Short-Term Effects of Nitrate-Rich Green Leafy Vegetables on Blood Pressure and Arterial Stiffness in Individuals with High-Normal Blood Pressure. Free Radic. Biol. Med..

[38] Jovanovski E., Bosco L., Khan K., Au-Yeung F., Ho H., Zurbau A., Jenkins A.L., Vuksan V. (2015). Effect of Spinach, a High Dietary Nitrate Source, on Arterial Stiffness and Related Hemodynamic Measures: A Randomized, Controlled Trial in Healthy Adults. Clin. Nutr. Res..

[39] Aatola H., Koivistoinen T., Hutri-Kähönen N., Juonala M., Mikkilä V., Lehtimäki T., Viikari J.S.A., Raitakari O.T., Kähönen M. (2010). Lifetime Fruit and Vegetable Consumption and Arterial Pulse Wave Velocity in Adulthood. Circulation.

[40] Liu S., Liu F.C., Li J.X., Huang K.Y., Yang X.L., Chen J.C., Cao J., Chen S.F., Huang J.F., Shen C. (2023). Association between Fruit and Vegetable Intake and Arterial Stiffness: The China-PAR Project. Biomed. Environ. Sci..

[41] Mustafa A.M., Abouelenein D., Acquaticci L., Alessandroni L., Angeloni S., Borsetta G., Caprioli G., Nzekoue F.K., Sagratini G., Vittori S. (2022). Polyphenols, Saponins and Phytosterols in Lentils and Their Health Benefits: An Overview. Pharmaceuticals.

[42] Rasouli H., Farzaei M.H., Khodarahmi R. (2017). Polyphenols and Their Benefits: A Review. Int. J. Food Prop..

[43] Park K. (2023). The Role of Dietary Phytochemicals: Evidence from Epidemiological Studies. Nutrients.

[44] Yamagata K., Tagami M., Yamori Y. (2015). Dietary Polyphenols Regulate Endothelial Function and Prevent Cardiovascular Disease. Nutrition.

[45] Bazzano L.A., Thompson A.M., Tees M.T., Nguyen C.H., Winham D.M. (2011). Non-Soy Legume Consumption Lowers Cholesterol Levels: A Meta-Analysis of Randomized Controlled Trials. Nutr. Metab. Cardiovasc. Dis..

[46] Khan K., Jovanovski E., Ho H.V.T., Marques A.C.R., Zurbau A., Mejia S.B., Sievenpiper J.L., Vuksan V. (2018). The Effect of Viscous Soluble Fiber on Blood Pressure: A Systematic Review and Meta-Analysis of Randomized Controlled Trials. Nutr. Metab. Cardiovasc. Dis..

[47] Chen Z., Ahmed M., Ha V., Jefferson K., Malik V., Ribeiro P.A.B., Zuchinali P., Drouin-Chartier J.-P. (2022). Dairy Product Consumption and Cardiovascular Health: A Systematic Review and Meta-Analysis of Prospective Cohort Studies. Adv. Nutr..

[48] Qin L.-Q., Xu J.-Y., Han S.-F., Zhang Z.-L., Zhao Y.-Y., Szeto I.M. (2015). Dairy Consumption and Risk of Cardiovascular Disease: An Updated Meta-Analysis of Prospective Cohort Studies. Asia Pac. J. Clin. Nutr..

[49] Zhang M., Dong X., Huang Z., Li X., Zhao Y., Wang Y., Zhu H., Fang A., Giovannucci E.L. (2023). Cheese Consumption and Multiple Health Outcomes: An Umbrella Review and Updated Meta-Analysis of Prospective Studies. Adv. Nutr..

[50] Ribeiro A., Mill J., Cade N., Velasquez-Melendez G., Matos S., Molina M. (2018). Associations of Dairy Intake with Arterial Stiffness in Brazilian Adults: The Brazilian Longitudinal Study of Adult Health (ELSA-Brasil). Nutrients.

[51] Diez-Fernández A., Álvarez-Bueno C., Martínez-Vizcaíno V., Sotos-Prieto M., Recio-Rodríguez J., Cavero-Redondo I. (2019). Total Dairy, Cheese and Milk Intake and Arterial Stiffness: A Systematic Review and Meta-Analysis of Cross-Sectional Studies. Nutrients.

[52] Bitok E., Sabaté J. (2018). Nuts and Cardiovascular Disease. Prog. Cardiovasc. Dis..

[53] Sarapis K., Thomas C.J., Hoskin J., George E.S., Marx W., Mayr H.L., Kennedy G., Pipingas A., Willcox J.C., Prendergast L.A. (2020). The Effect of High Polyphenol Extra Virgin Olive Oil on Blood Pressure and Arterial Stiffness in Healthy Australian Adults: A Randomized, Controlled, Cross-Over Study. Nutrients.

[54] Sanders T.A., Hall W.L., Maniou Z., Lewis F., Seed P.T., Chowienczyk P.J. (2011). Effect of Low Doses of Long-Chain N−3 PUFAs on Endothelial Function and Arterial Stiffness: A Randomized Controlled Trial. Am. J. Clin. Nutr..

[55] Kasliwal R.R., Bansal M., Mehrotra R., Yeptho K.P., Trehan N. (2015). Effect of Pistachio Nut Consumption on Endothelial Function and Arterial Stiffness. Nutrition.

[56] Neale E.P., Tapsell L.C., Guan V., Batterham M.J. (2017). The Effect of Nut Consumption on Markers of Inflammation and Endothelial Function: A Systematic Review and Meta-Analysis of Randomised Controlled Trials. BMJ Open.

[57] Xiao Y., Huang W., Peng C., Zhang J., Wong C., Kim J.H., Yeoh E., Su X. (2018). Effect of Nut Consumption on Vascular Endothelial Function: A Systematic Review and Meta-Analysis of Randomized Controlled Trials. Clin. Nutr..

[58] Shi W., Huang X., Schooling C.M., Zhao J.V. (2023). Red Meat Consumption, Cardiovascular Diseases, and Diabetes: A Systematic Review and Meta-Analysis. Eur. Heart J..

[59] Zhong V.W., Van Horn L., Greenland P., Carnethon M.R., Ning H., Wilkins J.T., Lloyd-Jones D.M., Allen N.B. (2020). Associations of Processed Meat, Unprocessed Red Meat, Poultry, or Fish Intake With Incident Cardiovascular Disease and All-Cause Mortality. JAMA Intern. Med..

[60] Acosta-Navarro J.C., Oki A.M., Antoniazzi L., Bonfim M.A.C., Hong V., Gaspar M.C.D.A., Sandrim V.C., Nogueira A. (2019). Consumption of Animal-Based and Processed Food Associated with Cardiovascular Risk Factors and Subclinical Atherosclerosis Biomarkers in Men. Rev. Assoc. Med. Bras..

[61] Borela V.L., de Alencar E.R., Mendonça M.A., Han H., Raposo A., Ariza-Montes A., Araya-Castillo L., Zandonadi R.P. (2022). Influence of Different Cooking Methods on Fillet Steak Physicochemical Characteristics. Int. J. Environ. Res. Public Health.

[62] Smiljanec K., Mbakwe A.U., Ramos-Gonzalez M., Mesbah C., Lennon S.L. (2020). Associations of Ultra-Processed and Unprocessed/Minimally Processed Food Consumption with Peripheral and Central Hemodynamics and Arterial Stiffness in Young Healthy Adults. Nutrients.

[63] Kondo K., Morino K., Nishio Y., Kondo M., Nakao K., Nakagawa F., Ishikado A., Sekine O., Yoshizaki T., Kashiwagi A. (2014). A Fish-Based Diet Intervention Improves Endothelial Function in Postmenopausal Women with Type 2 Diabetes Mellitus: A Randomized Crossover Trial. Metabolism.

[64] Wang M., Wang Z., Lee Y., Lai H.T.M., De Oliveira Otto M.C., Lemaitre R.N., Fretts A., Sotoodehnia N., Budoff M., Didonato J.A. (2022). Dietary Meat, Trimethylamine N-Oxide-Related Metabolites, and Incident Cardiovascular Disease Among Older Adults: The Cardiovascular Health Study. Arterioscler. Thromb. Vasc. Biol..

[65] Pierce G.L., Roy S.J., Gimblet C.J. (2021). The Gut-Arterial Stiffness Axis: Is TMAO a Novel Target to Prevent Age-Related Aortic Stiffening?. Hypertension.

[66] Azad B.J., Heshmati J., Daneshzad E., Palmowski A. (2021). Effects of Coffee Consumption on Arterial Stiffness and Endothelial Function: A Systematic Review and Meta-Analysis of Randomized Clinical Trials. Crit. Rev. Food. Sci. Nutr..

[67] Vlachopoulos C.V., Vyssoulis G.G., Alexopoulos N.A., Zervoudaki A.I., Pietri P.G., Aznaouridis K.A., Stefanadis C. (2007). Effect of Chronic Coffee Consumption on Aortic Stiffness and Wave Reflections in Hypertensive Patients. Eur. J. Clin. Nutr..

[68] Uemura H., Katsuura-Kamano S., Yamaguchi M., Nakamoto M., Hiyoshi M., Arisawa K. (2013). Consumption of Coffee, Not Green Tea, Is Inversely Associated with Arterial Stiffness in Japanese Men. Eur. J. Clin. Nutr..

[69] Pavão T.P., Chemello D., Ferigollo A., Saffi M.A.L., Moresco R.N., Stein C.D.S., Emanuelli T., Somacal S., Moriguchi E.H., Badimon L. (2022). Acute Effect of Coffee on Arterial Stiffness and Endothelial Function in Overweight and Obese Individuals: A Randomized Clinical Trial. Clin. Nutr. ESPEN.

[70] Noguchi K., Matsuzaki T., Sakanashi M., Hamadate N., Uchida T., Kina-Tanada M., Kubota H., Nakasone J., Sakanashi M., Ueda S. (2015). Effect of Caffeine Contained in a Cup of Coffee on Microvascular Function in Healthy Subjects. J. Pharmacol. Sci..

[71] Buscemi S., Verga S., Batsis J.A., Donatelli M., Tranchina M.R., Belmonte S., Mattina A., Re A., Cerasola G. (2010). Acute Effects of Coffee on Endothelial Function in Healthy Subjects. Eur. J. Clin. Nutr..

[72] Del Giorno R., Scanzio S., De Napoli E., Stefanelli K., Gabutti S., Troiani C., Gabutti L. (2022). Habitual Coffee and Caffeinated Beverages Consumption Is Inversely Associated with Arterial Stiffness and Central and Peripheral Blood Pressure. Int. J. Food Sci. Nutr..

[73] Higashi Y. (2019). Coffee and Endothelial Function: A Coffee Paradox?. Nutrients.

[74] Papaioannou T.G., Karatzi K., Karatzis E., Papamichael C., Lekakis J.P. (2005). Acute Effects of Caffeine on Arterial Stiffness, Wave Reflections, and Central Aortic Pressures. Am. J. Hypertens..

[75] Hendriks H.F.J. (2020). Alcohol and Human Health: What Is the Evidence?. Annu. Rev. Food Sci. Technol..

[76] Hwang C.-L., Muchira J., Hibner B.A., Phillips S.A., Piano M.R. (2022). Alcohol Consumption: A New Risk Factor for Arterial Stiffness?. Cardiovasc. Toxicol..

[77] Del Giorno R., Maddalena A., Bassetti S., Gabutti L. (2022). Association between Alcohol Intake and Arterial Stiffness in Healthy Adults: A Systematic Review. Nutrients.

[78] The Lancet Rheumatology (2023). Alcohol and Health: All, None, or Somewhere in-Between?. Lancet Rheumatol..

[79] Gonzalez-Sanchez J., Garcia-Ortiz L., Rodriguez-Sanchez E., Maderuelo-Fernandez J.A., Tamayo-Morales O., Lugones-Sanchez C., Recio-Rodriguez J.I., Gomez-Marcos M.A. (2020). The Relationship Between Alcohol Consumption With Vascular Structure and Arterial Stiffness in the Spanish Population: EVA Study. Alcohol Clin. Exp. Res..

[80] Sasaki S., Yoshioka E., Saijo Y., Kita T., Okada E., Tamakoshi A., Kishi R. (2013). Relation between Alcohol Consumption and Arterial Stiffness: A Cross-Sectional Study of Middle-Aged Japanese Women and Men. Alcohol.

[81] Hrelia S., Di Renzo L., Bavaresco L., Bernardi E., Malaguti M., Giacosa A. (2023). Moderate Wine Consumption and Health: A Narrative Review. Nutrients.

[82] Chiva-blanch G., Badimon L. (2020). Benefits and Risks of Moderate Alcohol Consumption on Cardiovascular Disease: Current Findings and Controversies. Nutrients.

[83] Wiles N.J., Lingford-Hughes A., Daniel J., Hickman M., Farrell M., Macleod J., Haynes J.C., Skapinakis P., Araya R., Lewis G. (2007). Socio-Economic Status in Childhood and Later Alcohol Use: A Systematic Review. Addiction.

[84] Collins S.E. (2016). Associations Between Socioeconomic Factors and Alcohol Outcomes. Alcohol Res..

[85] Hung H.C., Merchant A., Willett W., Ascherio A., Rosner B.A., Rimm E., Joshipura K.J. (2003). The Association between Fruit and Vegetable Consumption and Peripheral Arterial Disease. Epidemiology.

[86] Kondo K., Morino K., Nishio Y., Ishikado A., Arima H., Nakao K., Nakagawa F., Nikami F., Sekine O., Nemoto K.I. (2017). Fiber-Rich Diet with Brown Rice Improves Endothelial Function in Type 2 Diabetes Mellitus: A Randomized Controlled Trial. PLoS ONE.

[87] Brownlee I.A., Moore C., Chatfield M., Richardson D.P., Ashby P., Kuznesof S.A., Jebb S.A., Seal C.J. (2010). Markers of Cardiovascular Risk Are Not Changed by Increased Whole-Grain Intake: The WHOLEheart Study, a Randomised, Controlled Dietary Intervention. Br. J. Nutr..

[88] Vlachopoulos C., Panagiotakos D., Ioakeimidis N., Dima I., Stefanadis C. (2005). Chronic Coffee Consumption Has a Detrimental Effect on Aortic Stiffness and Wave Reflections. Am. J. Clin. Nutr..

[89] Puri V., Nagpal M., Singh I., Singh M., Dhingra G.A., Huanbutta K., Dheer D., Sharma A., Sangnim T. (2022). A Comprehensive Review on Nutraceuticals: Therapy Support and Formulation Challenges. Nutrients.

[90] Webb A.J., Patel N., Loukogeorgakis S., Okorie M., Aboud Z., Misra S., Rashid R., Miall P., Deanfield J., Benjamin N. (2008). Acute Blood Pressure Lowering, Vasoprotective, and Antiplatelet Properties of Dietary Nitrate via Bioconversion to Nitrite. Hypertension.

[91] Grassi D., Desideri G., Di Giosia P., De Feo M., Fellini E., Cheli P., Ferri L., Ferri C. (2013). Tea, Flavonoids, and Cardiovascular Health: Endothelial Protection. Am. J. Clin. Nutr..

[92] Shannon O.M., Clifford T., Seals D.R., Craighead D.H., Rossman M.J. (2022). Nitric Oxide, Aging and Aerobic Exercise: Sedentary Individuals to Master’s Athletes. Nitric Oxide.

[93] Walker M.A., Bailey T.G., McIlvenna L., Allen J.D., Green D.J., Askew C.D. (2019). Acute Dietary Nitrate Supplementation Improves Flow Mediated Dilatation of the Superficial Femoral Artery in Healthy Older Males. Nutrients.

[94] Floyd C.N., Lidder S., Hunt J., Omar S.A., McNeill K., Webb A.J. (2019). Acute Interaction between Oral Glucose (75 g as Lucozade) and Inorganic Nitrate: Decreased Insulin Clearance, but Lack of Blood Pressure-lowering. Br. J. Clin. Pharmacol..

[95] Joris P.J., Mensink R.P. (2013). Beetroot Juice Improves in Overweight and Slightly Obese Men Postprandial Endothelial Function after Consumption of a Mixed Meal. Atherosclerosis.

[96] Mayra S.T., Johnston C.S., Sweazea K.L. (2019). High-Nitrate Salad Increased Plasma Nitrates/Nitrites and Brachial Artery Flow-Mediated Dilation in Postmenopausal Women: A Pilot Study. Nutr. Res..

[97] Zafeiridis A., Triantafyllou A., Papadopoulos S., Koletsos N., Touplikioti P., Zafeiridis A.S., Gkaliagkousi E., Dipla K., Douma S. (2019). Dietary Nitrate Improves Muscle Microvascular Reactivity and Lowers Blood Pressure at Rest and during Isometric Exercise in Untreated Hypertensives. Microcirculation.

[98] Morselli F., Faconti L., Mills C.E., Morant S., Chowienczyk P.J., Yeung J.A., Cavarape A., Cruickshank J.K., Webb A.J. (2021). Dietary Nitrate Prevents Progression of Carotid Subclinical Atherosclerosis through Blood Pressure-independent Mechanisms in Patients with or at Risk of Type 2 Diabetes Mellitus. Br. J. Clin. Pharmacol..

[99] Kapil V., Milsom A.B., Okorie M., Maleki-Toyserkani S., Akram F., Rehman F., Arghandawi S., Pearl V., Benjamin N., Loukogeorgakis S. (2010). Inorganic Nitrate Supplementation Lowers Blood Pressure in Humans. Hypertension.

[100] Bahrami L.S., Arabi S.M., Feizy Z., Rezvani R. (2021). The Effect of Beetroot Inorganic Nitrate Supplementation on Cardiovascular Risk Factors: A Systematic Review and Meta-Regression of Randomized Controlled Trials. Nitric Oxide.

[101] Alasmari A.M., Alsulayyim A.S., Alghamdi S.M., Philip K.E.J., Buttery S.C., Banya W.A.S., Polkey M.I., Armstrong P.C., Rickman M.J., Warner T.D. (2024). Oral Nitrate Supplementation Improves Cardiovascular Risk Markers in COPD: ON-BC, a Randomised Controlled Trial. Eur. Respir. J..

[102] Dyakova E.Y., Kapilevich L.V., Shylko V.G., Popov S.V., Anfinogenova Y. (2015). Physical Exercise Associated with NO Production: Signaling Pathways and Significance in Health and Disease. Front. Cell Dev. Biol..

[103] Nyawose S., Naidoo R., Naumovski N., McKune A.J. (2022). The Effects of Consuming Amino Acids L-Arginine, L-Citrulline (and Their Combination) as a Beverage or Powder, on Athletic and Physical Performance: A Systematic Review. Beverages.

[104] Mirvish S.S. (1991). The Significance for Human Health of Nitrate, Nitrite and N-Nitroso Compounds. Nitrate Contamination.

[105] Boink A.B.T.J., Vleeming W., Dormans J.A.M.A., Speijers G.J.A. (1999). Effects of Nitrates and Nitrites in Experimental Animals. Managing Risks of Nitrates to Humans and the Environment.

[106] Kotopoulou S., Zampelas A., Magriplis E. (2022). Dietary Nitrate and Nitrite and Human Health: A Narrative Review by Intake Source. Nutr. Rev..

[107] Nitrate/Nitrite Toxicity: What Are the Health Effects from Exposure to Nitrates and Nitrites? Environmental Medicine. ATSDR. https://www.atsdr.cdc.gov/csem/nitrate-nitrite/health_effects.html.

[108] Ward M.H., Jones R.R., Brender J.D., de Kok T.M., Weyer P.J., Nolan B.T., Villanueva C.M., van Breda S.G. (2018). Drinking Water Nitrate and Human Health: An Updated Review. Int. J. Environ. Res. Public Health.

[109] Shannon O.M., Easton C., Shepherd A.I., Siervo M., Bailey S.J., Clifford T. (2021). Dietary Nitrate and Population Health: A Narrative Review of the Translational Potential of Existing Laboratory Studies. BMC Sports Sci. Med. Rehabil..

[110] Lau C.W.Z., Hamers A.J.P., Rathod K.S., Shabbir A., Cooper J., Primus C.P., Davies C., Mathur A., Moon J.C., Kapil V. (2020). Randomised, Double-Blind, Placebo-Controlled Clinical Trial Investigating the Effects of Inorganic Nitrate in Hypertension-Induced Target Organ Damage: Protocol of the NITRATE-TOD Study in the UK. BMJ Open.

[111] Salehi B., Mishra A., Nigam M., Sener B., Kilic M., Sharifi-Rad M., Fokou P., Martins N., Sharifi-Rad J. (2018). Resveratrol: A Double-Edged Sword in Health Benefits. Biomedicines.

[112] Zhou D.-D., Luo M., Huang S.-Y., Saimaiti A., Shang A., Gan R.-Y., Li H.-B. (2021). Effects and Mechanisms of Resveratrol on Aging and Age-Related Diseases. Oxid. Med. Cell. Longev..

[113] Du L., Chen E., Wu T., Ruan Y., Wu S. (2019). Resveratrol Attenuates Hydrogen Peroxide-Induced Aging through Upregulation of Autophagy in Human Umbilical Vein Endothelial Cells. Drug Des. Devel. Ther..

[114] Rajapakse A.G., Yepuri G., Carvas J.M., Stein S., Matter C.M., Scerri I., Ruffieux J., Montani J.-P., Ming X.-F., Yang Z. (2011). Hyperactive S6K1 Mediates Oxidative Stress and Endothelial Dysfunction in Aging: Inhibition by Resveratrol. PLoS ONE.

[115] Parsamanesh N., Asghari A., Sardari S., Tasbandi A., Jamialahmadi T., Xu S., Sahebkar A. (2021). Resveratrol and Endothelial Function: A Literature Review. Pharmacol. Res..

[116] Wong R.H.X., Berry N.M., Coates A.M., Buckley J.D., Bryan J., Kunz I., Howe P.R.C. (2013). Chronic Resveratrol Consumption Improves Brachial Flow-Mediated Dilatation in Healthy Obese Adults. J. Hypertens..

[117] Gonçalinho G.H.F., Roggerio A., Goes M.F.D.S., Avakian S.D., Leal D.P., Strunz C.M.C., Mansur A.D.P. (2021). Comparison of Resveratrol Supplementation and Energy Restriction Effects on Sympathetic Nervous System Activity and Vascular Reactivity: A Randomized Clinical Trial. Molecules.

[118] Diaz M., Avila A., Degens H., Coeckelberghs E., Vanhees L., Cornelissen V., Azzawi M. (2020). Acute Resveratrol Supplementation in Coronary Artery Disease: Towards Patient Stratification. Scand. Cardiovasc. J..

[119] Wong R.H.X., Howe P.R.C., Buckley J.D., Coates A.M., Kunz I., Berry N.M. (2011). Acute Resveratrol Supplementation Improves Flow-Mediated Dilatation in Overweight/Obese Individuals with Mildly Elevated Blood Pressure. Nutr. Metab. Cardiovasc. Dis..

[120] Brown K., Theofanous D., Britton R.G., Aburido G., Pepper C., Sri Undru S., Howells L. (2024). Resveratrol for the Management of Human Health: How Far Have We Come? A Systematic Review of Resveratrol Clinical Trials to Highlight Gaps and Opportunities. Int. J. Mol. Sci..

[121] Walle T. (2011). Bioavailability of Resveratrol. Ann. N. Y. Acad. Sci..

[122] Panche A.N., Diwan A.D., Chandra S.R. (2016). Flavonoids: An Overview. J. Nutr. Sci..

[123] Bondonno C.P., Croft K.D., Ward N., Considine M.J., Hodgson J.M. (2015). Dietary Flavonoids and Nitrate: Effects on Nitric Oxide and Vascular Function. Nutr. Rev..

[124] Lilamand M., Kelaiditi E., Guyonnet S., Antonelli Incalzi R., Raynaud-Simon A., Vellas B., Cesari M. (2014). Flavonoids and Arterial Stiffness: Promising Perspectives. Nutr. Metab. Cardiovasc. Dis..

[125] Natarajan T.D., Ramasamy J.R., Palanisamy K. (2019). Nutraceutical Potentials of Synergic Foods: A Systematic Review. J. Ethn. Foods.

[126] Hooper L., Kroon P.A., Rimm E.B., Cohn J.S., Harvey I., Le Cornu K.A., Ryder J.J., Hall W.L., Cassidy A. (2008). Flavonoids, Flavonoid-Rich Foods, and Cardiovascular Risk: A Meta-Analysis of Randomized Controlled Trials. Am. J. Clin. Nutr..

[127] Heiss C., Sansone R., Karimi H., Krabbe M., Schuler D., Rodriguez-Mateos A., Kraemer T., Cortese-Krott M.M., Kuhnle G.G.C., Spencer J.P.E. (2015). Impact of Cocoa Flavanol Intake on Age-Dependent Vascular Stiffness in Healthy Men: A Randomized, Controlled, Double-Masked Trial. Age.

[128] Sansone R., Rodriguez-Mateos A., Heuel J., Falk D., Schuler D., Wagstaff R., Kuhnle G.G.C., Spencer J.P.E., Schroeter H., Merx M.W. (2015). Cocoa Flavanol Intake Improves Endothelial Function and Framingham Risk Score in Healthy Men and Women: A Randomised, Controlled, Double-Masked Trial: The Flaviola Health Study. Br. J. Nutr..

[129] Gröne M., Sansone R., Höffken P., Horn P., Rodriguez-Mateos A., Schroeter H., Kelm M., Heiss C. (2020). Cocoa Flavanols Improve Endothelial Functional Integrity in Healthy Young and Elderly Subjects. J. Agric. Food Chem..

[130] Garcia-Yu I.A., Garcia-Ortiz L., Gomez-Marcos M.A., Rodriguez-Sanchez E., Agudo-Conde C., Gonzalez-Sanchez J., Maderuelo-Fernandez J.A., Recio-Rodriguez J.I. (2020). Effects of Cocoa-Rich Chocolate on Blood Pressure, Cardiovascular Risk Factors, and Arterial Stiffness in Postmenopausal Women: A Randomized Clinical Trial. Nutrients.

[131] Odai T., Terauchi M., Kato K., Hirose A., Miyasaka N. (2019). Effects of Grape Seed Proanthocyanidin Extract on Vascular Endothelial Function in Participants with Prehypertension: A Randomized, Double-Blind, Placebo-Controlled Study. Nutrients.

[132] Barona J., Aristizabal J.C., Blesso C.N., Volek J.S., Fernandez M.L. (2012). Grape Polyphenols Reduce Blood Pressure and Increase Flow-Mediated Vasodilation in Men with Metabolic Syndrome. J. Nutr..

[133] Vaisman N., Niv E. (2015). Daily Consumption of Red Grape Cell Powder in a Dietary Dose Improves Cardiovascular Parameters: A Double Blind, Placebo-Controlled, Randomized Study. Int. J. Food Sci. Nutr..

[134] Draijer R., de Graaf Y., Slettenaar M., de Groot E., Wright C. (2015). Consumption of a Polyphenol-Rich Grape-Wine Extract Lowers Ambulatory Blood Pressure in Mildly Hypertensive Subjects. Nutrients.

[135] Siasos G., Tousoulis D., Kokkou E., Oikonomou E., Kollia M.-E., Verveniotis A., Gouliopoulos N., Zisimos K., Plastiras A., Maniatis K. (2014). Favorable Effects of Concord Grape Juice on Endothelial Function and Arterial Stiffness in Healthy Smokers. Am. J. Hypertens..

[136] Micek A., Godos J., Del Rio D., Galvano F., Grosso G. (2021). Dietary Flavonoids and Cardiovascular Disease: A Comprehensive Dose-Response Meta-Analysis. Mol. Nutr. Food Res..

[137] Duffy S.J., Keaney J.F., Holbrook M., Gokce N., Swerdloff P.L., Frei B., Vita J.A. (2001). Short- and Long-Term Black Tea Consumption Reverses Endothelial Dysfunction in Patients With Coronary Artery Disease. Circulation.

[138] Nagaya N., Yamamoto H., Uematsu M., Itoh T., Nakagawa K., Miyazawa T., Kangawa K., Miyatake K. (2004). Green Tea Reverses Endothelial Dysfunction in Healthy Smokers. Heart.

[139] Rees A., Dodd G.F., Spencer J.P.E. (2018). The Effects of Flavonoids on Cardiovascular Health: A Review of Human Intervention Trials and Implications for Cerebrovascular Function. Nutrients.

[140] Ezzati M., Yousefi B., Velaei K., Safa A. (2020). A Review on Anti-Cancer Properties of Quercetin in Breast Cancer. Life Sci..

[141] Deepika, Maurya P.K. (2022). Health Benefits of Quercetin in Age-Related Diseases. Molecules.

[142] Huang H., Liao D., Dong Y., Pu R. (2020). Effect of Quercetin Supplementation on Plasma Lipid Profiles, Blood Pressure, and Glucose Levels: A Systematic Review and Meta-Analysis. Nutr. Rev..

[143] Serban M., Sahebkar A., Zanchetti A., Mikhailidis D.P., Howard G., Antal D., Andrica F., Ahmed A., Aronow W.S., Muntner P. (2016). Effects of Quercetin on Blood Pressure: A Systematic Review and Meta-Analysis of Randomized Controlled Trials. J. Am. Heart Assoc..

[144] Choi E.-Y., Lee H., Woo J.S., Jang H.H., Hwang S.J., Kim H.S., Kim W.-S., Kim Y.-S., Choue R., Cha Y.-J. (2015). Effect of Onion Peel Extract on Endothelial Function and Endothelial Progenitor Cells in Overweight and Obese Individuals. Nutrition.

[145] Dehghani F., Vafa M., Ebrahimkhani A., Găman M.A., Sezavar Seyedi Jandaghi S.H. (2023). Effects of Quercetin Supplementation on Endothelial Dysfunction Biomarkers and Depression in Post-Myocardial Infarction Patients: A Double-Blind, Placebo-Controlled, Randomized Clinical Trial. Clin. Nutr. ESPEN.

[146] Boots A.W., Li H., Schins R.P.F., Duffin R., Heemskerk J.W.M., Bast A., Haenen G.R.M.M. (2007). The Quercetin Paradox. Toxicol. Appl. Pharmacol..

[147] Andres S., Pevny S., Ziegenhagen R., Bakhiya N., Schäfer B., Hirsch-Ernst K.I., Lampen A. (2018). Safety Aspects of the Use of Quercetin as a Dietary Supplement. Mol. Nutr. Food Res..

[148] Liu Y., Luo X., Yang C., Yang T., Zhou J., Shi S. (2016). Impact of Quercetin-Induced Changes in Drug-Metabolizing Enzyme and Transporter Expression on the Pharmacokinetics of Cyclosporine in Rats. Mol. Med. Rep..

[149] Wang Y.H., Chao P.D.L., Hsiu S.L., Wen K.C., Hou Y.C. (2004). Lethal Quercetin-Digoxin Interaction in Pigs. Life Sci..

[150] Bedada S.K., Neerati P. (2018). Evaluation of the Effect of Quercetin Treatment on CYP2C9 Enzyme Activity of Diclofenac in Healthy Human Volunteers. Phytother. Res..

[151] Woodward C.J., Deyo Z.M., Donahue K.E., Deal A.M., Hawes E.M. (2014). Clinically Relevant Interaction between Warfarin and Scuppernongs, a Quercetin Containing Muscadine Grape: Continued Questions Surrounding Flavonoid-Induced Warfarin Interactions. BMJ Case Rep..

[152] Patel R., Stine A., Zitko K. (2022). Enhanced Anticoagulant Effect of Warfarin When Co-Administered With Quercetin. J. Pharm. Technol..

[153] El-Saber Batiha G., Beshbishy A.M., Ikram M., Mulla Z.S., Abd El-Hack M.E., Taha A.E., Algammal A.M., Ali Elewa Y.H. (2020). The Pharmacological Activity, Biochemical Properties, and Pharmacokinetics of the Major Natural Polyphenolic Flavonoid: Quercetin. Foods.

[154] Bernatoniene J., Kopustinskiene D. (2018). The Role of Catechins in Cellular Responses to Oxidative Stress. Molecules.

[155] Liguori I., Russo G., Curcio F., Bulli G., Aran L., Della-Morte D., Gargiulo G., Testa G., Cacciatore F., Bonaduce D. (2018). Oxidative Stress, Aging, and Diseases. Clin. Interv. Aging.

[156] Kuriyama S., Shimazu T., Ohmori K., Kikuchi N., Nakaya N., Nishino Y., Tsubono Y., Tsuji I. (2006). Green Tea Consumption and Mortality Due to Cardiovascular Disease, Cancer, and All Causes in Japan. JAMA.

[157] de Koning Gans J.M., Uiterwaal C.S.P.M., van der Schouw Y.T., Boer J.M.A., Grobbee D.E., Verschuren W.M.M., Beulens J.W.J. (2010). Tea and Coffee Consumption and Cardiovascular Morbidity and Mortality. Arterioscler. Thromb. Vasc. Biol..

[158] Cheng T.O. (2003). Why Did Green Tea Not Protect against Coronary Artery Disease but Protect against Myocardial Infarction?. Am. J. Cardiol..

[159] Mangels D.R., Mohler E.R. (2017). Catechins as Potential Mediators of Cardiovascular Health. Arterioscler. Thromb. Vasc. Biol..

[160] Jochmann N., Lorenz M., Krosigk A.V., Martus P., Böhm V., Baumann G., Stangl K., Stangl V. (2008). The Efficacy of Black Tea in Ameliorating Endothelial Function Is Equivalent to That of Green Tea. Br. J. Nutr..

[161] Grassi D., Mulder T.P., Draijer R., Desideri G., Molhuizen H.O., Ferri C. (2009). Black Tea Consumption Dose-Dependently Improves Flow-Mediated Dilation in Healthy Males. J. Hypertens..

[162] Schreuder T.H.A., Eijsvogels T.M.H., Greyling A., Draijer R., Hopman M.T.E., Thijssen D.H.J. (2014). Effect of Black Tea Consumption on Brachial Artery Flow-Mediated Dilation and Ischaemia–Reperfusion in Humans. Appl. Physiol. Nutr. Metab..

[163] Sapper T.N., Mah E., Ahn-Jarvis J., McDonald J.D., Chitchumroonchokchai C., Reverri E.J., Vodovotz Y., Bruno R.S. (2016). A Green Tea-Containing Starch Confection Increases Plasma Catechins without Protecting against Postprandial Impairments in Vascular Function in Normoglycemic Adults. Food Funct..

[164] Hodgson J.M., Puddey I.B., Burke V., Watts G.F., Beilin L.J. (2002). Regular Ingestion of Black Tea Improves Brachial Artery Vasodilator Function. Clin. Sci..

[165] Shafabakhsh R., Milajerdi A., Reiner Ž., Kolahdooz F., Amirani E., Mirzaei H., Barekat M., Asemi Z. (2020). The Effects of Catechin on Endothelial Function: A Systematic Review and Meta-Analysis of Randomized Controlled Trials. Crit. Rev. Food Sci. Nutr..

[166] Younes M., Aggett P., Aguilar F., Crebelli R., Dusemund B., Filipič M., Frutos M.J., Galtier P., Gott D., Gundert-Remy U. (2018). Scientific Opinion on the Safety of Green Tea Catechins. EFSA J..

[167] Cheng T.O. (2004). Is Green Tea Better Than Black Tea in Reducing Atherosclerosis?. Circulation.

[168] Riccioni G., Bazzano L.A. (2008). Antioxidant Plasma Concentration and Supplementation in Carotid Intima Media Thickness. Expert Rev. Cardiovasc. Ther..

[169] Tsang C., Smail N.F., Almoosawi S., McDougall G.J.M., Al-Dujaili E.A.S. (2018). Antioxidant Rich Potato Improves Arterial Stiffness in Healthy Adults. Plant Foods Hum. Nutr..

[170] Fairlie-Jones L., Davison K., Fromentin E., Hill A. (2017). The Effect of Anthocyanin-Rich Foods or Extracts on Vascular Function in Adults: A Systematic Review and Meta-Analysis of Randomised Controlled Trials. Nutrients.

[171] Arisi T.O.P., Gorski F., Eibel B., Barbosa E., Boll L., Waclawovsky G., Lehnen A.M. (2023). Dietary Intake of Anthocyanins Improves Arterial Stiffness, but Not Endothelial Function, in Volunteers with Excess Weight: A Randomized Clinical Trial. Phytother. Res..

[172] Arenas-Jal M., Suñé-Negre J.M., García-Montoya E. (2020). Coenzyme Q10 Supplementation: Efficacy, Safety, and Formulation Challenges. Compr. Rev. Food Sci. Food Saf..

[173] Hargreaves I.P., Mantle D. (2019). Coenzyme Q10 Supplementation in Fibrosis and Aging. Reviews on Biomarker Studies in Aging and Anti-Aging Research.

[174] Gao J., Xu Y., Jia H., Zhang L., Cao X., Zuo X., Cai G., Chen X. (2021). Associations of Coenzyme Q10 with Endothelial Function in Hemodialysis Patients. Nephrology.

[175] Kawashima C., Matsuzawa Y., Konishi M., Akiyama E., Suzuki H., Sato R., Nakahashi H., Kikuchi S., Kimura Y., Maejima N. (2020). Ubiquinol Improves Endothelial Function in Patients with Heart Failure with Reduced Ejection Fraction: A Single-Center, Randomized Double-Blind Placebo-Controlled Crossover Pilot Study. Am. J. Cardiovasc. Drugs.

[176] Hamilton S.J., Chew G.T., Watts G.F. (2009). Coenzyme Q10 Improves Endothelial Dysfunction in Statin-Treated Type 2 Diabetic Patients. Diabetes Care.

[177] Gao L., Mao Q., Cao J., Wang Y., Zhou X., Fan L. (2012). Effects of Coenzyme Q10 on Vascular Endothelial Function in Humans: A Meta-Analysis of Randomized Controlled Trials. Atherosclerosis.

[178] Shalansky S., Lynd L., Richardson K., Ingaszewski A., Kerr C. (2007). Risk of Warfarin-Related Bleeding Events and Supratherapeutic International Normalized Ratios Associated with Complementary and Alternative Medicine: A Longitudinal Analysis. Pharmacotherapy.

[179] Baskaran R., Shanmugam S., Nagayya-Sriraman S., Kim J.H., Jeong T.C., Yong C.S., Choi H.G., Yoo B.K. (2008). The Effect of Coenzyme Q10 on the Pharmacokinetic Parameters of Theophylline. Arch. Pharm. Res..

[180] Lund E.L., Quistorff B., Spang-Thomsen M., Kristjansen P.E.G. (1998). Effect of Radiation Therapy on Small-Cell Lung Cancer Is Reduced by Ubiquinone Intake. Folia Microbiol..

[181] Zhou Q., Zhou S., Chan E. (2005). Effect of Coenzyme Q10 on Warfarin Hydroxylation in Rat and Human Liver Microsomes. Curr. Drug Metab..

[182] Houston M. (2013). Nutrition and Nutraceutical Supplements for the Treatment of Hypertension: Part III. J. Clin. Hypertens..

[183] Fischer A., Schmelzer C., Rimbach G., Niklowitz P., Menke T., Döring F. (2011). Association between Genetic Variants in the Coenzyme Q10 Metabolism and Coenzyme Q10 Status in Humans. BMC Res. Notes.

[184] Karimian M.S., Pirro M., Johnston T.P., Majeed M., Sahebkar A. (2017). Curcumin and Endothelial Function: Evidence and Mechanisms of Protective Effects. Curr. Pharm. Des..

[185] Santos-Parker J.R., Strahler T.R., Bassett C.J., Bispham N.Z., Chonchol M.B., Seals D.R. (2017). Curcumin Supplementation Improves Vascular Endothelial Function in Healthy Middle-Aged and Older Adults by Increasing Nitric Oxide Bioavailability and Reducing Oxidative Stress. Aging.

[186] Alidadi M., Sahebkar A., Eslami S., Vakilian F., Jarahi L., Alinezhad-Namaghi M., Arabi S.M., Vakili S., Tohidinezhad F., Nikooiyan Y. (2021). The Effect of Curcumin Supplementation on Pulse Wave Velocity in Patients with Metabolic Syndrome: A Randomized, Double-Blind, Placebo-Controlled Trial. Adv. Exp. Med. Biol..

[187] Dehzad M.J., Ghalandari H., Askarpour M. (2024). Curcumin/Turmeric Supplementation Could Improve Blood Pressure and Endothelial Function: A Grade-Assessed Systematic Review and Dose-Response Meta-Analysis of Randomized Controlled Trials. Clin. Nutr. ESPEN.

[188] Racz L.Z., Racz C.P., Pop L.C., Tomoaia G., Mocanu A., Barbu I., Sárközi M., Roman I., Avram A., Tomoaia-Cotisel M. (2022). Strategies for Improving Bioavailability, Bioactivity, and Physical-Chemical Behavior of Curcumin. Molecules.

[189] Tabanelli R., Brogi S., Calderone V. (2021). Improving Curcumin Bioavailability: Current Strategies and Future Perspectives. Pharmaceutics.

[190] Hewlings S.J., Kalman D.S. (2017). Curcumin: A Review of Its Effects on Human Health. Foods.

[191] Bahramsoltani R., Rahimi R., Farzaei M.H. (2017). Pharmacokinetic Interactions of Curcuminoids with Conventional Drugs: A Review. J. Ethnopharmacol..

[192] Lee Y.S., Park J.W., Joo M., Moon S., Kim K., Kim M.G. (2023). Effects of Omega-3 Fatty Acids on Flow-Mediated Dilatation and Carotid Intima Media Thickness: A Meta-Analysis. Curr. Atheroscler. Rep..

[193] Sawada T., Tsubata H., Hashimoto N., Takabe M., Miyata T., Aoki K., Yamashita S., Oishi S., Osue T., Yokoi K. (2016). Effects of 6-Month Eicosapentaenoic Acid Treatment on Postprandial Hyperglycemia, Hyperlipidemia, Insulin Secretion Ability, and Concomitant Endothelial Dysfunction among Newly-Diagnosed Impaired Glucose Metabolism Patients with Coronary Artery Disease. An Open Label, Single Blinded, Prospective Randomized Controlled Trial. Cardiovasc. Diabetol..

[194] Meital L.T., Schulze K., Magee R., O’Donnell J., Jha P., Meital C.Y., Donkin R., Bailey T.G., Askew C.D., Russell F.D. (2020). Long Chain Omega-3 Polyunsaturated Fatty Acids Improve Vascular Stiffness in Abdominal Aortic Aneurysm: A Randomized Controlled Trial. Nutrients.

[195] Maruyama K., Khairunnisa S., Saito I., Tanigawa T., Tomooka K., Minato-Inokawa S., Sano M., Takakado M., Kawamura R., Takata Y. (2022). Association of Fish and Omega-3 Fatty Acid Intake with Carotid Intima-Media Thickness in Middle-Aged to Elderly Japanese Men and Women: The Toon Health Study. Nutrients.

[196] Casanova M.A., Medeiros F., Trindade M., Cohen C., Oigman W., Neves M.F. (2017). Omega-3 Fatty Acids Supplementation Improves Endothelial Function and Arterial Stiffness in Hypertensive Patients with Hypertriglyceridemia and High Cardiovascular Risk. J. Am. Soc. Hypertens..

[197] Żebrowska A., Mizia-Stec K., Mizia M., Gąsior Z., Poprzęcki S. (2015). Omega-3 Fatty Acids Supplementation Improves Endothelial Function and Maximal Oxygen Uptake in Endurance-trained Athletes. Eur. J. Sport Sci..

[198] Wang Q., Liang X., Wang L., Lu X., Huang J., Cao J., Li H., Gu D. (2012). Effect of Omega-3 Fatty Acids Supplementation on Endothelial Function: A Meta-Analysis of Randomized Controlled Trials. Atherosclerosis.

[199] Petersen M.M., Eschen R.B., Aardestrup I., Obel T., Schmidt E.B. (2010). Flow-Mediated Vasodilation and Dietary Intake of n-3 Polyunsaturated Acids in Healthy Subjects. Cell. Mol. Biol..

[200] O’Mahoney L.L., Dunseath G., Churm R., Holmes M., Boesch C., Stavropoulos-Kalinoglou A., Ajjan R.A., Birch K.M., Orsi N.M., Mappa G. (2020). Omega-3 Polyunsaturated Fatty Acid Supplementation versus Placebo on Vascular Health, Glycaemic Control, and Metabolic Parameters in People with Type 1 Diabetes: A Randomised Controlled Preliminary Trial. Cardiovasc. Diabetol..

[201] Chang J.P.C., Tseng P.T., Zeng B.S., Chang C.H., Su H., Chou P.H., Su K.P. (2023). Safety of Supplementation of Omega-3 Polyunsaturated Fatty Acids: A Systematic Review and Meta-Analysis of Randomized Controlled Trials. Adv. Nutr..

[202] Ambati R., Phang S.-M., Ravi S., Aswathanarayana R. (2014). Astaxanthin: Sources, Extraction, Stability, Biological Activities and Its Commercial Applications—A Review. Mar. Drugs.

[203] Bjørklund G., Gasmi A., Lenchyk L., Shanaida M., Zafar S., Mujawdiya P.K., Lysiuk R., Antonyak H., Noor S., Akram M. (2022). The Role of Astaxanthin as a Nutraceutical in Health and Age-Related Conditions. Molecules.

[204] Bjørklund G., Dadar M., Martins N., Chirumbolo S., Goh B.H., Smetanina K., Lysiuk R. (2018). Brief Challenges on Medicinal Plants: An Eye-Opening Look at Ageing-Related Disorders. Basic Clin. Pharmacol. Toxicol..

[205] Ma B., Lu J., Kang T., Zhu M., Xiong K., Wang J. (2022). Astaxanthin Supplementation Mildly Reduced Oxidative Stress and Inflammation Biomarkers: A Systematic Review and Meta-Analysis of Randomized Controlled Trials. Nutr. Res..

[206] Landi F., Martone A.M., Salini S., Zazzara B., Calvani R., Marzetti E., Nesci A., Di Giorgio A., Giupponi B., Santoro L. (2019). Effects of a New Combination of Medical Food on Endothelial Function and Lipid Profile in Dyslipidemic Subjects: A Pilot Randomized Trial. BioMed Res. Int..

[207] Birudaraju D., Cherukuri L., Kinninger A., Chaganti B.T., Shaikh K., Hamal S., Flores F., Roy S.K., Budoff M.J. (2020). A Combined Effect of Cavacurcumin, Eicosapentaenoic Acid (Omega-3s), Astaxanthin and Gamma –Linoleic Acid (Omega-6) (CEAG) in Healthy Volunteers- a Randomized, Double-Blind, Placebo-Controlled Study. Clin. Nutr. ESPEN.

[208] Bjørklund G., Shanaida M., Lysiuk R., Antonyak H., Klishch I., Shanaida V., Peana M. (2022). Selenium: An Antioxidant with a Critical Role in Anti-Aging. Molecules.

[209] Swart R., Schutte A.E., van Rooyen J.M., Mels C.M.C. (2019). Selenium and Large Artery Structure and Function: A 10-Year Prospective Study. Eur. J. Nutr..

[210] Djaoudene O., Romano A., Bradai Y.D., Zebiri F., Ouchene A., Yousfi Y., Amrane-Abider M., Sahraoui-Remini Y., Madani K. (2023). A Global Overview of Dietary Supplements: Regulation, Market Trends, Usage during the COVID-19 Pandemic, and Health Effects. Nutrients.

[211] Han J., Zhao C., Cai J., Liang Y. (2020). Comparative Efficacy of Vitamin Supplements on Prevention of Major Cardiovascular Disease: Systematic Review with Network Meta-Analysis. Complement. Ther. Clin. Pract..

[212] Shechter M., Sharir M., Paul M.J., James L., Burton F., Noel S.C., Merz B. (2000). Oral Magnesium Therapy Improves Endothelial Function in Patients with Coronary Artery Disease. Circulation.

[213] Joris P.J., Plat J., Bakker S.J.L., Mensink R.P. (2016). Long-Term Magnesium Supplementation Improves Arterial Stiffness in Overweight and Obese Adults: Results of a Randomized, Double-Blind, Placebo-Controlled Intervention Trial. Am. J. Clin. Nutr..

[214] Schutten J.C., Joris P.J., Groendijk I., Eelderink C., Groothof D., van der Veen Y., Westerhuis R., Goorman F., Danel R.M., de Borst M.H. (2022). Effects of Magnesium Citrate, Magnesium Oxide, and Magnesium Sulfate Supplementation on Arterial Stiffness: A Randomized, Double-Blind, Placebo-Controlled Intervention Trial. J. Am. Heart Assoc..

[215] Marques B.C.A.A., Klein M.R.S.T., da Cunha M.R., de Souza Mattos S., de Paula Nogueira L., de Paula T., Corrêa F.M., Oigman W., Neves M.F. (2020). Effects of Oral Magnesium Supplementation on Vascular Function: A Systematic Review and Meta-Analysis of Randomized Controlled Trials. High Blood Press. Cardiovasc. Prev..

[216] Gröber U. (2019). Magnesium and Drugs. Int. J. Mol. Sci..

[217] Dinicolantonio J.J., Liu J., O’Keefe J.H. (2018). Magnesium for the Prevention and Treatment of Cardiovascular Disease. Open Heart.

[218] Pak C.Y.C., Nicar M.J., Britton F. (1983). Clinical Experience with Sodium Cellulose Phosphate. World J. Urol..

[219] Crippa G., Sverzellati E., Giorgi-Pierfranceschi M., Carrara G.C. (1999). Magnesium and Cardiovascular Drugs: Interactions and Therapeutic Role. Ann. Ital. Med. Int..

[220] Arayne M.S., Sultana N., Hussain F. (2005). Interactions between Ciprofloxacin and Antacids--Dissolution and Adsorption Studies. Drug Metabol. Drug Interact..

[221] Fukunaga M. (2001). Risedronate: A Review of Its Pharmacological Properties and Clinical Use in Resorptive Bone Disease. Drugs.

[222] Maruyama K., Eshak E.S., Kinuta M., Nagao M., Cui R., Imano H., Ohira T., Iso H. (2019). Association between Vitamin B Group Supplementation with Changes in % Flow-Mediated Dilatation and Plasma Homocysteine Levels: A Randomized Controlled Trial. J. Clin. Biochem. Nutr..

[223] Kwok T., Chook P., Qiao M., Tam L., Poon Y.K.P., Ahuja A.T., Woo J., Celermajer D.S., Woo K.S. (2012). Vitamin B-12 Supplementation Improves Arterial Function in Vegetarians with Subnormal Vitamin B-12 Status. J. Nutr. Health Aging.

[224] de Bree A., van Mierlo L.A., Draijer R. (2007). Folic Acid Improves Vascular Reactivity in Humans: A Meta-Analysis of Randomized Controlled Trials. Am. J. Clin. Nutr..

[225] Zamani M., Rezaiian F., Saadati S., Naseri K., Ashtary-Larky D., Yousefi M., Golalipour E., Clark C.C.T., Rastgoo S., Asbaghi O. (2023). The Effects of Folic Acid Supplementation on Endothelial Function in Adults: A Systematic Review and Dose-Response Meta-Analysis of Randomized Controlled Trials. Nutr. J..

[226] Bokayeva K., Jamka M., Banaszak M., Makarewicz-Bukowska A., Adamczak A., Chrobot M., Janicka A., Jaworska N., Walkowiak J. (2023). The Effect of Folic Acid Supplementation on Endothelial Function and Arterial Stiffness Markers in Adults: A Systematic Review and Meta-Analysis. Healthcare.

[227] Seet R.C.S., Lee C.-Y.J., Lim E.C.H., Quek A.M.L., Huang H., Huang S.H., Looi W.F., Long L.H., Halliwell B. (2011). Oral Zinc Supplementation Does Not Improve Oxidative Stress or Vascular Function in Patients with Type 2 Diabetes with Normal Zinc Levels. Atherosclerosis.

[228] Rasool A.H.G., Rehman A., Yusuf W.N.W., Rahman A.R.A. (2003). Vitamin E and Its Effect on Arterial Stiffness in Postmenopausal Women—A Randomized Controlled Trial. Int. J. Clin. Pharmacol. Ther..

[229] Skyrme-Jones R.A.P., O’Brien R.C., Berry K.L., Meredith I.T. (2000). Vitamin E Supplementation Improves Endothelial Function in Type I Diabetes Mellitus: A Randomized, Placebo-Controlled Study. J. Am. Coll. Cardiol..

[230] Ashor A.W., Siervo M., Lara J., Oggioni C., Afshar S., Mathers J.C. (2015). Effect of Vitamin C and Vitamin E Supplementation on Endothelial Function: A Systematic Review and Meta-Analysis of Randomised Controlled Trials. Br. J. Nutr..

[231] Mullan B.A., Young I.S., Fee H., McCance D.R. (2002). Ascorbic Acid Reduces Blood Pressure and Arterial Stiffness in Type 2 Diabetes. Hypertension.

[232] Rodríguez A.J., Scott D., Srikanth V., Ebeling P. (2016). Effect of Vitamin D Supplementation on Measures of Arterial Stiffness: A Systematic Review and Meta-Analysis of Randomized Controlled Trials. Clin. Endocrinol..

[233] Witham M.D., Adams F., Kabir G., Kennedy G., Belch J.J.F., Khan F. (2013). Effect of Short-Term Vitamin D Supplementation on Markers of Vascular Health in South Asian Women Living in the UK--a Randomised Controlled Trial. Atherosclerosis.

[234] Witham M.D., Dove F.J., Sugden J.A., Doney A.S., Struthers A.D. (2012). The Effect of Vitamin D Replacement on Markers of Vascular Health in Stroke Patients—A Randomised Controlled Trial. Nutr. Metab. Cardiovasc. Dis..

[235] Kumar V., Yadav A.K., Lal A., Kumar V., Singhal M., Billot L., Gupta K.L., Banerjee D., Jha V. (2017). A Randomized Trial of Vitamin D Supplementation on Vascular Function in CKD. J. Am. Soc. Nephrol..

[236] Sluyter J.D., Camargo C.A., Stewart A.W., Waayer D., Lawes C.M.M., Toop L., Khaw K.T., Thom S.A.M.G., Hametner B., Wassertheurer S. (2017). Effect of Monthly, High-Dose, Long-Term Vitamin D Supplementation on Central Blood Pressure Parameters: A Randomized Controlled Trial Substudy. J. Am Heart Assoc..

[237] Lips P., de Jongh R.T., van Schoor N.M. (2021). Trends in Vitamin D Status Around the World. JBMR Plus.

[238] Rebelos E., Tentolouris N., Jude E. (2023). The Role of Vitamin D in Health and Disease: A Narrative Review on the Mechanisms Linking Vitamin D with Disease and the Effects of Supplementation. Drugs.

[239] Hathcock J.N., Shao A., Vieth R., Heaney R. (2007). Risk Assessment for Vitamin D. Am. J. Clin. Nutr..

[240] Vieth R. (1999). Vitamin D Supplementation, 25-Hydroxyvitamin D Concentrations, and Safety. Am. J. Clin. Nutr..

[241] Vieth R. (2007). Vitamin D Toxicity, Policy, and Science. J. Bone Miner. Res..

[242] Vlasschaert C., Goss C.J., Pilkey N.G., McKeown S., Holden R.M. (2020). Vitamin K Supplementation for the Prevention of Cardiovascular Disease: Where Is the Evidence? A Systematic Review of Controlled Trials. Nutrients.

[243] Geng C., Huang L., Pu L., Feng Y. (2022). Effects of Vitamin K Supplementation on Vascular Calcification in Chronic Kidney Disease: A Systematic Review and Meta-Analysis of Randomized Controlled Trials. Front. Nutr..

[244] Blanch N., Clifton P.M., Keogh J.B. (2014). Postprandial Effects of Potassium Supplementation on Vascular Function and Blood Pressure: A Randomised Cross-over Study. Nutr. Metab. Cardiovasc. Dis..

[245] Tang X., Wu B., Luo Y., Peng L., Chen Y., Zhu J., Peng C., Li S., Liu J. (2017). Effect of Potassium Supplementation on Vascular Function: A Meta-Analysis of Randomized Controlled Trials. Int. J. Cardiol..

[246] D’Elia L., Cappuccio F.P., Masulli M., La Fata E., Rendina D., Galletti F. (2023). Effect of Potassium Supplementation on Endothelial Function: A Systematic Review and Meta-Analysis of Intervention Studies. Nutrients.

[247] Harris E., Rowsell R., Pipingas A., Macpherson H. (2016). No Effect of Multivitamin Supplementation on Central Blood Pressure in Healthy Older People: A Randomized Controlled Trial. Atherosclerosis.

[248] Van Dijk S.C., Enneman A.W., Swart K.M.A., Van Wijngaarden J.P., Ham A.C., Brouwer-Brolsma E.M., Van Der Zwaluw N.L., Blom H.J., Feskens E.J., Geleijnse J.M. (2015). Effects of 2-Year Vitamin B12 and Folic Acid Supplementation in Hyperhomocysteinemic Elderly on Arterial Stiffness and Cardiovascular Outcomes within the B-PROOF Trial. J. Hypertens..

[249] Knapen M.H.J., Braam L.A.J.L.M., Drummen N.E., Bekers O., Hoeks A.P.G., Vermeer C. (2015). Menaquinone-7 Supplementation Improves Arterial Stiffness in Healthy Postmenopausal Women. A Double-Blind Randomised Clinical Trial. Thromb. Haemost..

[250] Dalan R., Goh L.L., Lim C.J., Seneviratna A., Liew H., Seow C.J., Xia L., Chew D.E.K., Leow M.K.S., Boehm B.O. (2020). Impact of Vitamin E Supplementation on Vascular Function in Haptoglobin Genotype Stratified Diabetes Patients (EVAS Trial): A Randomised Controlled Trial. Nutr. Diabetes.

[251] Akbari M., Tamtaji O.R., Lankarani K.B., Tabrizi R., Dadgostar E., Kolahdooz F., Jamilian M., Mirzaei H., Asemi Z. (2019). The Effects of Resveratrol Supplementation on Endothelial Function and Blood Pressures Among Patients with Metabolic Syndrome and Related Disorders: A Systematic Review and Meta-Analysis of Randomized Controlled Trials. High Blood Press. Cardiovasc. Prev..

[252] Mohammadipoor N., Shafiee F., Rostami A., Kahrizi M.S., Soleimanpour H., Ghodsi M., Ansari M.J., Bokov D.O., Jannat B., Mosharkesh E. (2022). Resveratrol Supplementation Efficiently Improves Endothelial Health: A Systematic Review and Meta-Analysis of Randomized Controlled Trials. Phytother. Res..

[253] Jalili F., Moradi S., Talebi S., Mehrabani S., Ghoreishy S.M., Wong A., Jalalvand A.R., Kermani M.A.H., Jalili C., Jalili F. (2024). The Effects of Citrus Flavonoids Supplementation on Endothelial Function: A Systematic Review and Dose-Response Meta-Analysis of Randomized Clinical Trials. Phytother. Res..

[254] Tamtaji O.R., Milajerdi A., Dadgostar E., Kolahdooz F., Chamani M., Amirani E., Mirzaei H., Asemi Z. (2019). The Effects of Quercetin Supplementation on Blood Pressures and Endothelial Function Among Patients with Metabolic Syndrome and Related Disorders: A Systematic Review and Meta-Analysis of Randomized Controlled Trials. Curr. Pharm. Des..

[255] Jeong H.S., Kim S., Hong S.J., Choi S.C., Choi J.H., Kim J.H., Park C.Y., Cho J.Y., Lee T.B., Kwon J.W. (2016). Black Raspberry Extract Increased Circulating Endothelial Progenitor Cells and Improved Arterial Stiffness in Patients with Metabolic Syndrome: A Randomized Controlled Trial. J. Med. Food.

[256] Zhu Y., Xia M., Yang Y., Liu F., Li Z., Hao Y., Mi M., Jin T., Ling W. (2011). Purified Anthocyanin Supplementation Improves Endothelial Function via NO-CGMP Activation in Hypercholesterolemic Individuals. Clin. Chem..

[257] Grimes D.A., Schulz K.F. (2002). Bias and Causal Associations in Observational Research. Lancet.

